# Senescence Cell Induction Methods Display Diverse Metabolic Reprogramming and Reveal an Underpinning Serine/Taurine Reductive Metabolic Phenotype

**DOI:** 10.1111/acel.70127

**Published:** 2025-06-18

**Authors:** Domenica Berardi, Gillian Farrell, Abdullah AlSultan, Ashley McCulloch, Nicole M. Hall, Rui Pedro Pereira Sousa, Melanie Jimenez, Colin Selman, Ilaria Bellantuono, Caroline H. Johnson, Zahra Rattray, Nicholas J. W. Rattray

**Affiliations:** ^1^ Department of Environmental Health Sciences, Yale School of Public Health Yale University New Haven Connecticut USA; ^2^ Strathclyde Institute of Pharmacy and Biomedical Science University of Strathclyde Glasgow UK; ^3^ Faculty of Pharmacy Kuwait University Safat Kuwait; ^4^ Wolfson Wohl Cancer Research Centre, School of Cancer Sciences University of Glasgow Glasgow UK; ^5^ Department of Biomedical Engineering University of Strathclyde Glasgow UK; ^6^ School of Molecular Biosciences University of Glasgow Glasgow UK; ^7^ Department of Oncology and Metabolism, Healthy Lifespan Institute and MRC–Arthritis Research UK Centre for Integrated Research Into Musculoskeletal Ageing University of Sheffield Sheffield UK; ^8^ Strathclyde Centre for Molecular Bioscience University of Strathclyde Glasgow UK

**Keywords:** aging, isotope labeling, metabolomics, proteomics, rheo‐morphology, senescence

## Abstract

The relationship between in vitro senescence cell induction and intracellular biomolecular dysregulation is still poorly understood. In this study, we have found that a range of metabolic subphenotypes exists and is dependent on the induction method that is used. To develop understanding of these subphenotypes, we developed and employed a novel bioanalytical pipeline integrating untargeted metabolomics, label‐free proteomics, and stable isotope tracing alongside cellular deformability measurements and established senescence biomarkers. Initially, standard senescent markers indicated all induction methods were consistent by showing elevated SA‐β‐Gal expression, p21 levels, and γH2AX DNA damage markers alongside a decrease in Ki67 and an increase in shape, volume, and deformability. However, when probed at the metabolic and protein levels, all senescence models indicated both shared and unique biomolecular responses. A metabolic shift toward reductive pathways (driven by serine and taurine rewiring) and impaired proteostasis was an observed shared response. These findings suggest that targeting metabolic redox circuits, alongside serine and taurine metabolic processes, presents novel therapeutic strategies for addressing senescence and aging. But importantly, alongside this general shift, we found that significant metabolic and proteomic heterogeneity also exists across different senescence induction methods. This demonstrates that the method of senescence induction significantly influences cell metabolic and proteomic profiles. Critically, methods of senescence induction are not interchangeable, and careful consideration is needed when choosing between different induction methods and when comparing cellular phenotypes across different in vitro senescence experiments.

## Graphical abstract

This study reveals that in vitro senescence induction methods significantly influence intracellular biomolecular profiles, showing both shared and unique metabolic responses. A novel bioanalytical pipeline was developed to explore these subphenotypes, highlighting the importance of targeting metabolic redox circuits and serine/taurine pathways for therapeutic strategies against senescence and aging.

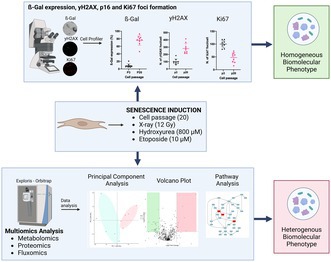

## 1 Introduction

The systemic accumulation of biomolecular damage at the cellular level plays a central role in the aging process. A key contributor to this process is the senescent cellular phenotype, identified by irreversible cell growth arrest, substantial morphological changes, and broad biomolecular dysregulation (Campesi 2012). Associated hallmarks of senescence include telomere shortening, DNA damage, the production of reactive oxygen species (ROS), associated mitochondrial dysregulation, and overall rewiring of carbon source metabolism (Hernandez‐Segura et al. 2018). The identification and characterization of senescent cells within models of aging is an area of intense research due to growing evidence that the removal of these cells restores a healthier aging phenotype, making them viable therapeutic targets (Di Micco et al. 2021; Zhang et al. 2022).

Multiple studies have shown that the accumulation of senescent cells in tissues disrupts their structure and alters their functions, thereby contributing to aging and the development of related diseases such as neurodegeneration (Khosla et al. 2020), endocrine diseases (Erusalimsky and Kurz 2005), cardiovascular disease (Mehdizadeh et al. 2022) and cancer (Campisi et al. 2011). In these pathologies, senescent cells have been demonstrated to contribute to degenerative changes through structural enlargement/stiffening (Tilton et al. 2024), telomere shortening (Rossiello et al. 2022), mitochondrial dysfunction (Miwa et al. 2022), impaired autophagy (Fang et al. 2021), and the secretion of a milieu of biomolecules that constitute the inflammatory mediating senescence‐associated secretory phenotype (SASP) (González‐Gualda et al. 2021). Alongside a concerted effort to profile SASP, a variety of in vitro methods targeting biomolecular changes in senescent cells are currently used for their identification, characterization, and mechanistic understanding (Lawless et al. 2010). These methods are often used in a combinatorial fashion and include primary cell markers such as altered cell cycle regulators p16/Rb and p21/p53 expression (Itahana et al. 2007) biomolecular changes like senescence‐associated β‐galactosidase (SA‐β‐Gal) activity (Sedelnikova et al. 2004) and accumulation of DNA double‐strand breaks marked by γH2AX (Malaquin et al. 2019). Secondary markers, including increased ROS and SASP (Hernandez‐Segura et al. 2017), SASP‐mediated lipid dysregulation (Zeng et al. 2024), alongside metabolic rewiring in glucose/glutamine metabolism (Wiley and Campesi 2021) leading to the accumulation of advanced glycation end‐products (AGEs) (Chaudhuri et al. 2018) further complement these analyses. Critically, the expression of these biomarkers within in vitro senescence models has been shown to vary depending on the specific stimuli used to induce a senescence response (Noren Hooten and Evans 2017). Therefore, effective intracellular biomolecular characterization of specific induced senescence phenotypes is required to assess the factors that contribute to associated dysregulation and to allow for appropriate senescence model selection for future studies.

Mass spectrometry‐based metabolite/protein profiling and stable isotope tracing are powerful analytical approaches that aid in identifying mechanisms of disease, potentially leading to the development of accurate and sensitive alternative detection strategies for senescence subtypes. The application of these technologies, alongside cell deformability studies, holds huge potential in the field of aging because they can provide a large set of functional metabolites and proteins that depict the complexity of biomolecular dysregulation associated with the aging process—while identifying new therapeutic targets in the process. In this study, a novel multiomics morpho‐rheological platform has been developed and used to define the baseline phenotypic and biomolecular changes of different senescent‐induced cellular subtypes of HFF‐1 fibroblast cells. These models were generated through the processes of replicative passage, exposure to DNA damage, production of ROS, and epigenetic alterations through irradiation and treatment with senescence‐inducing chemicals to reflect the different modes of senescent cells commonly used by senescence researchers (Al Sultan et al. 2024).

Subsequent analysis and integration of metabolomics, proteomics, and rheological datasets are employed for the identification of senescence profiles along with their inflammatory phenotype. Further stable isotope tracing of ^13^C_6_‐glucose and ^13^C_5_,^15^N_2_‐glutamine enabled delineation of the direction of the metabolic pathways contributing to the rewiring of senescence‐associated metabolism, identifying a senescence gluconeogenesis phenotype where energy metabolism is directed toward amino acid sources.

## 2 Materials and Methods

### 2.1 Cell Line, Chemicals, and Treatment

All cell culture reagents were obtained from Gibco (Thermo Fisher Scientific). The human foreskin fibroblast cell line (HFF‐1, ATCC SCRC‐1041) was purchased and maintained in Dulbecco's modified Eagle's medium (DMEM, high‐glucose) supplemented with 10% v/v FBS (Invitrogen), 1% v/v nonessential amino acids (Thermo Fisher Scientific), and 1% v/v penicillin–streptomycin (Invitrogen). Cells were maintained in a prehumified atmosphere containing 5% v/v CO_2_ at 37°C.

Hydroxyurea (Sigma Fisher Scientific) was prepared as a 10 mM stock in water. Etoposide (Merck) was prepared as a 1 mM stock solution in DMSO. All drug stocks were aliquoted and stored at 20°C until use. Crystal violet (Fisher Scientific) stain was prepared with 20% methanol (Alfa Aesar) and 2% sucrose (VWR Life Science) and stored at room temperature. Primary antibodies for γH2AX, Ki67, and p21 (Cell Signaling Technologies) were used for immunostaining alongside Alexa Fluor 488‐conjugated secondary antibody (Fisher Scientific).

Cells were passaged multiple times in culture (upon which passages 3–20 were compared) and passage 3 was also treated with ascending doses of irradiation (1–12 Gy for 1 week), hydroxyurea (0–1,000 μM for 2 weeks), or etoposide (0–50 μM for 1 week) until markers of senescence appeared (growth arrest, increase in cell size and senescence‐associated SA‐ß‐Gal expression).

### 2.2 Senescence‐Associated ß‐Galactosidase Staining

Cells were seeded in a 96‐well plate at a density of 4000 cells per well and incubated for a predefined period for each specific treatment type. Following incubation, SA‐β‐Gal activity was assessed using a senescence detection kit (ab65351, Abcam) as per the manufacturer's recommendations. Subsequently, cells were counterstained with 4′,6‐diamidino‐2‐phenylindole (DAPI) to stain their nuclei. Image acquisition was carried out using an Invitrogen EVOS Auto Imaging System (AMAFD1000‐Thermo Fisher Scientific) with a minimum of 100 cells imaged per treatment condition. SA‐β‐Gal‐stained cells were manually counted with ImageJ software v 1.54. The number of SA‐β‐Gal‐stained cells was normalized with the number of counted nuclei.

### 2.3 Immunostaining for γH2AX, Ki67, and p21

Foci immunodetection for γH2AX, Ki67, and p21 was performed in low and high passage cells 7 days after seeding for nonirradiated cells. Irradiated cells exposed to 12 Gy were measured at 7 days, and in nontreated and treated cells with hydroxyurea (800 μM) and etoposide (10 μM) for 14 and 7 days, respectively. Briefly, cell monolayers were fixed in chilled 4% w/v formaldehyde containing 2% w/v sucrose in phosphate‐buffered solution, followed by fixation in ice‐cold methanol (100% v/v). Subsequently, cells were permeabilized in 0.25% v/v Triton X‐100 in PBS, blocked with 5% v/v goat serum/5% w/v BSA, and immunoprobed with a primary rabbit anti‐γH2AX (1:800), anti‐P21 (1:800), or primary mouse anti‐Ki67 (1:800) antibody overnight at 4°C. Cell monolayers were treated with goat anti‐rabbit and anti‐mouse Alexa Fluor 488 conjugated secondary antibody (1:1000) and counterstained with DAPI. Image acquisition was carried out using an Invitrogen EVOS Auto Imaging System (AMAFD1000‐Thermo Fisher Scientific) with a minimum of 100 cells imaged per treatment condition. Resultant γH2AX foci, Ki‐67, and p21 labeled nuclei images were analyzed in CellProfiler (v.4.2.1.) using a modified version of the “speckle counting” and “percent positive” pipeline, respectively. Further thresholding settings for nuclei, γH2AX foci, and Ki‐67 labeled nuclei are indicated in Table 1.

**TABLE 1:** Thresholding parameters applied in CellProfiler for the detection of foci and labeled nuclei.

	DAPI	γH2AX	Ki‐67	p21
Diameter of objects (in pixel units)	Min: 8 Max: 80	Min: 1 Max: 40	Min: 5 Max: 200	Min: 5 Max: 200
Distinguish objects by	Shape	Intensity	Intensity	Intensity
Thresholding method	Minimum cross‐entropy	Minimum cross‐entropy	Minimum cross‐entropy	Minimum cross‐entropy
Lower and upper bounds on threshold	0.05, 1.00	0.00, 1.00	0.00, 1.00	0.00, 1.00

### 2.4 Deformability Assay

Morpho‐rheological analysis of different models of senescence using real‐time deformability cytometry. Cells were detached utilizing 0.05% (w/v) trypsin (Thermo Fisher) preceded by two chelating steps using 0.02 w/v EDTA (Thermo Fisher). Cells were suspended in Dulbecco's modified Eagle medium (DMEM) (VWR, 392‐0415) to neutralize trypsin. Cells were centrifuged at 220 RCF for 5 min and resuspended in Cell Carrier B (ZellMechanik, Dresden). Cell deformability was tested using a polydimethylsiloxane (PDMS) microfluidic chip with a channel of 30 × 30 μm^2^ profile (ZellMechanik, Dresden). Measurements were taken on both channel and reservoir.

### 2.5 Metabolomics and Stable Isotope Tracer Analysis

#### 2.5.1 Sample Preparation

HFF‐1 cells were seeded at a density of 2 × 10^6^ cells per well in six‐well clear polycarbonate plates, at passage 20 and before exposure to either 12 Gy irradiation or growth medium containing 800 μM hydroxyurea or 10 μM etoposide. For tracer experiments, after incubation, cells were pulsed with heavy glucose and/or glutamine isotopes dissolved in DMEM (A1443001 ThermoFisher Scientific) supplemented with 10% FBS, to concentrations of 25 mM ^13^C_6_‐glucose (110187‐42‐3, Cambridge Isotope Laboratories) and 4 mM ^13^C_5_,^15^N_2_‐glutamine (285978–14‐5, Cambridge Isotope Laboratories). After 24 h, the growth medium was aspirated from each well, centrifuged to remove cell debris, aliquoted, and stored at −80°C. Subsequently, treated and labeled cells were washed with prechilled PBS, with metabolites quenched and extracted in a final volume of 1.5 mL prechilled (−80°C) methanol:acetonitrile:water solvent (50:30:20). Resultant cell pellets were collected, flash frozen in liquid nitrogen, vortexed, and sonicated for 3 min in an ice‐water bath. This process was performed in triplicate. Extracts were centrifuged at 13,000 × g for 10 min at 4°C and pellets were retained for protein quantification using the Bradford assay (Pierce Coomassie Plus Bradford Assay Kit, Thermo Scientific). The resultant supernatant was collected and dried under vacuum for 10 h (Savant‐SPD121P). Dried metabolite pellets were subsequently reconstituted in acetonitrile:water (50:50) at volumes normalized to the relative Bradford assay‐derived protein content. Quality control (QC) samples were prepared by pooling samples across all control and treatment groups. Solvent blank (reconstitution buffer) and QC samples were inserted in the analytical batch.

#### 2.5.2 LC–MS/MS

Metabolite separation was performed on a binary Thermo Vanquish ultrahigh‐performance liquid chromatography system where 2 μL of the reconstituted cellular extract was injected onto a ZIC‐pHILIC column (MERCK—100 mm × 2.1 mm, particle size 5 μm). Blank and QC samples were injected after every five samples to assess the stability of the detecting system. The temperature of the column oven was maintained at 35°C, while the autosampler temperature was set at 5°C. For chromatographic separation, a consistent flow rate of 300 μL/min was used, where the mobile phase in positive and negative heated electrospray ionization mode (HESI^+/−^) was composed of buffer A (100% acetonitrile) and buffer B (20 mM ammonium formate in water with 0.1% formic acid). The elution gradient used for the chromatographic separation of metabolites is included in Table S1.

A high‐resolution Exploris 240‐Orbitrap mass spectrometer (Thermo Fisher Scientific) was used to perform a full scan and fragmentation analysis. Global operating parameters were set as follows: spray voltages of 3900 V in HESI +ve mode and 2500 V in HESI –ve mode. The temperature of the transfer tube was set at 320°C with a vaporizer temperature of 300°C. Sheath, aux gas, and sheath gas flow rates were set at 40, 10, and 1 Arb, respectively. A Top‐5 data‐dependent acquisition (DDA) was performed using the following parameter: survey scan range was 50–750 m/z with MS1 resolution of 60,000. Subsequent MS/MS scans were processed with a resolution of 30,000. High‐purity nitrogen was used as nebulizing and as the collision gas for higher energy collisional dissociation. Further details are included in Table S2.

#### 2.5.3 Data Processing

Raw data files obtained from Thermo Scientific Xcalibur TM software v4.2 were imported into Compound Discoverer 3.3 software where the “Untargeted Metabolomics with Statistics Detect Unknowns with ID Using Local Databases” feature was selected (all settings are provided in Table S3). The workflow analysis performed retention time (RT) alignment, unknown compound detection, predicted elemental compositions for all compounds, and removed features from the general chemical background (using blank samples). For the detection of compounds, mass and RT tolerance were set to 5 ppm and 0.3 min, respectively. The library search was conducted against an in‐house library of 425 pure chemical standards alongside the mz‐cloud database. A compound table was generated with a list of metabolites (known and unknown). Among them, compounds were selected according to fully matching mass, RT, and fragmentation spectra of the local database. The selected metabolites were then used to perform pathway and statistical analysis.

### 2.6 Proteomics Analysis

#### 2.6.1 Sample Preparation

HFF‐1 cells were seeded at a density of 2 × 10^6^ cells per well in six‐well plates, at passage 20 and before exposure to either 12 Gy irradiation or growth medium containing 800 μM hydroxyurea or 10 μM etoposide. After incubation, label‐free sample preparation for proteomics was carried out according to the EasyPep MS Sample Prep Kits (Thermo Fisher Scientific). Briefly, cells were lysed in 100 μL of lysis solution, homogenized, and centrifuged at 16,000 × g for 10 min. Following protein quantification, 100 μg of protein sample was reduced, alkylated, and digested with trypsin/Lys‐C protease mix. Subsequently, peptides were polished through a peptide‐clean‐up spin column and cleaned up with buffer solutions provided by the vendor to remove hydrophilic and hydrophobic contaminants. The clean peptide solution was then dried under vacuum for 10 h. Dried peptide extracts were reconstituted in 100 μL of 0.1% formic acid in water for LC–MS analysis.

#### 2.6.2 LC–MS/MS

Peptide separation was performed on a binary Thermo Vanquish ultrahigh‐performance liquid chromatography system where 10 μL of the cellular protein extract was injected onto an Acclaim PepMap 100 (150 × 1.0 mm, particle size 3 μm) and followed an in‐house microflow label‐free proteomics method (Krtolica et al. 2001). Blank and QC samples were injected after every five samples to assess the stability of the detecting system. The temperature of the column oven was maintained at 40°C while the autosampler temperature was set at 5°C. For chromatographic separation, a consistent flow rate of 50 μL/min was used where the mobile phase in positive heated electrospray ionization mode (HESI^+^) was composed of buffer A (water with 0.1% formic acid)) and buffer B (acetonitrile with 0.1% formic acid). The elution gradient used for the chromatographic separation of peptides is included in Table S4.

Global operating parameters for proteomics analysis were set as follows: spray voltages of 3400 V in HESI^+^ mode and 3000 in HESI mode. The temperature of the transfer tube was set at 320°C with a vaporizer temperature of 75°C. Sheath, aux gas, and sweep gas flow rates were set at 25, 5, and 0 Arb, respectively. DDA was performed using the following parameters: full scan range was 275–1500 m/z with an MS1 resolution of 120,000. A Top‐20 DDA was performed using parameters documented in Table S5.

Data processing—Raw data files obtained from Thermo Scientific Xcalibur 4.2 software were imported into Proteome Discoverer 3.0 for label‐free proteomics analysis, with details included in Table S6.

### 2.7 Joint Pathway Analysis

Joint pathway analysis was based on the 2024 KEGG Pathway API performed in the MetaboAnalyst software (https://dev.metaboanalyst.ca/Secure/pathinteg/IntegParamView.xhtml) and was achieved by integrating exome‐situated genes relative to identified proteins with the list of ID compounds and their associated Log_2_ fold change values. A full explanation of the method is explained in Ewald et al. 2024. The integration method combined both genes and metabolites into a single query and then was used to perform enrichment analysis through a hypergeometric test. The hypergeometric test defines the significance of the association between two sets of data (genes and metabolites) (Ramell et al. 2018). Finally, important nodes (compounds) were scored based on their betweenness centrality, and pathway analysis results were generated.

### 2.8 Statistical Analysis

For metabolomics and proteomics analysis, principal component analysis (PCA) was performed to reduce data dimensionality and determine the clustering of each sample group. Differential analysis was used to compare differences between control and treatment groups and plotted as a Volcano plot (log2‐fold change vs. −log10 *p* value). Peak areas were log_10_ transformed and *p* values were calculated for the sample group by two‐tailed *t*‐test assuming that all data were normally distributed. A *p* < 0.05 and a fold change of 1.5 were deemed to be statistically significant. For deformability analysis, data were visualized and extracted using ShapeOut2 (version 2.13.6). Statistical analysis was performed in IBM SPSS Statistics (version 28.0.0.0 (190)). Statistical significance was tested using an independent‐sample Mann–Whitney *U* test. Jensen divergence was applied for the area and deformability of different models of senescence, comparing distributions for control and test cells in the reservoir and channel.

## 3 Results

### 3.1 Induction of Senescence in Human Fibroblasts and Expression of Senescence‐Associated ß‐Galactosidase

HFF‐1 cells were used in this study owing to their previous widespread adoption as a model of replicative senescence across numerous tissues and organs (Pang et al. 2024; Schafer et al. 2017; Waters et al. 2018; Zhang et al. 2024). To design and classify the biomolecular differences of functional models of replicative and stress‐induced senescence, cells were passaged multiple times in culture (3–20 replicates) and, in parallel, the same cell line was treated with different doses of X‐rays (0–12 Gy), hydroxyurea (0–100 μM), or etoposide (0–50 μM). The level of senescence was optimized for each condition with a target cell viability of ~50% (Figure S1) and > 50% of SA‐β‐Gal‐positive cells in suboptimal conditions (pH < 6) (Figure S2). Upon optimization, cell passage 20, dosage of 12 Gy irradiation, 800 μM hydroxyurea, and 10 μM etoposide were used for subsequent imaging, morphology, and omics/tracer analyses to ensure an adequate number of cells (biomass) and the changes observed were not related to cell death dependent on cytotoxicity. Based on our results, senescence was induced across all the conditions examined. In particular, ~80% of cells were SA‐β‐Gal positive after 20 passages and treatment with 800 μM hydroxyurea, while cells treated with 12 Gy IR and 10 μM etoposide resulted in > 60% SA‐β‐Gal‐positive cells (Figure S3). Of note, the control samples for each condition (passage 3, 0 Gy IR, and 0 μM of hydroxyurea and etoposide) had a baseline percentage of SA‐β‐Gal‐positive cells which corresponds to < 20% in the passaged, irradiated, and etoposide‐treated cells after 1 week incubation, while ~30% in the hydroxyurea‐treated cells following 2 weeks incubation. These results show a baseline number of senescent cells exist under normal cell culture conditions and that SA‐β‐Gal expression is influenced also by the experimental incubation time.

### 3.2 Cellular Deformability and Size of Senescence‐Induced Phenotypes

**FIGURE 1:**
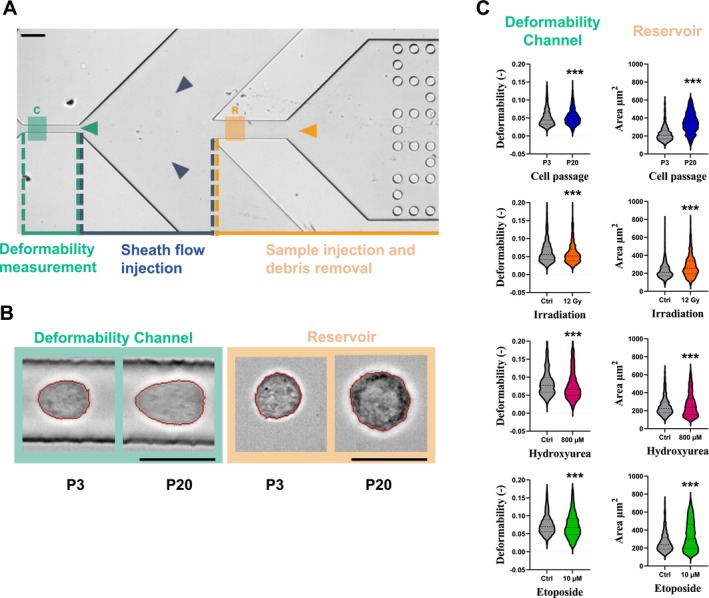
Morpho‐rheological analysis of different models of senescence using real‐time deformability cytometry. (A) Cells are injected into a microfluidic channel and pinched by a viscous sheath flow for deformability measurements. Arrows represent the direction of flow, and C (resp. R) represents the ROI for cell imaging in the deformability channel (resp. reservoir). Scale bar is 100 μm. (B) Images of single cells at early passage (EP) and late passage (LP) numbers in the deformability channel and reservoir. Red lines represent the automated cell contour detection for area and deformability measurements. Scale bars are 30 μm. (C) Area (right) measurement in the reservoir and deformation (left) measurement in the deformability channel (*n* ~ 1000 cells for each group) indicate significant differences in all senescence‐induced models.

Cell morphology analysis indicated that late passaged and chemically induced cells produce an increased surface area compared to their relative controls (Figure S4) which indicates a morphological change associated with elevated cellular stress. To investigate the morphological changes induced by each method, real‐time deformability cytometry was employed to measure alterations in cell volume and deformability (Figure 1). The design of the microfluidic chamber enabled the measurement of the initial shape of cells in the reservoir and their deformation in the deformability channel (Figure 1a,b). Corresponding measured cell area was significantly larger at passage 20, following exposure to 12 Gy irradiation, and in response to treatment with 800 μM hydroxyurea and 10 μM etoposide in comparison to controls (Figure 1c). The most significant increase in measured cell area was observed between early (P3) and late passaged cells (P20) with a measured mean area of 227.3 ± 84.7 μm^2^ for early passage and 347.7 ± 114.8 μm^2^ for late passage cells. HU treatment led to the smallest variation in size, with no significant difference compared to the control in the reservoir (mean area 251.2 ± 102.9 μm^2^ for 0 μM and 265.9 ± 130.7 μm^2^ for 800 μM) (Table S7). Significant differences were observed in the deformability channel for all tested conditions (Table S8). No significant differences were observed in mechanical deformability for passaged and irradiated in the reservoir (initial conditions). Significant differences were, however, observed in the reservoir for etoposide and hydroxyurea‐treated cells (Table S9). This supplies substantial evidence that different senescence induction methods lead to a broad range of mechanocellular phenotypes.

### 3.3 Senescence‐Associated Molecular Markers of DNA Damage (γH2AX) and Cell Cycle Activity (Ki67 and p21)

**FIGURE 2:**
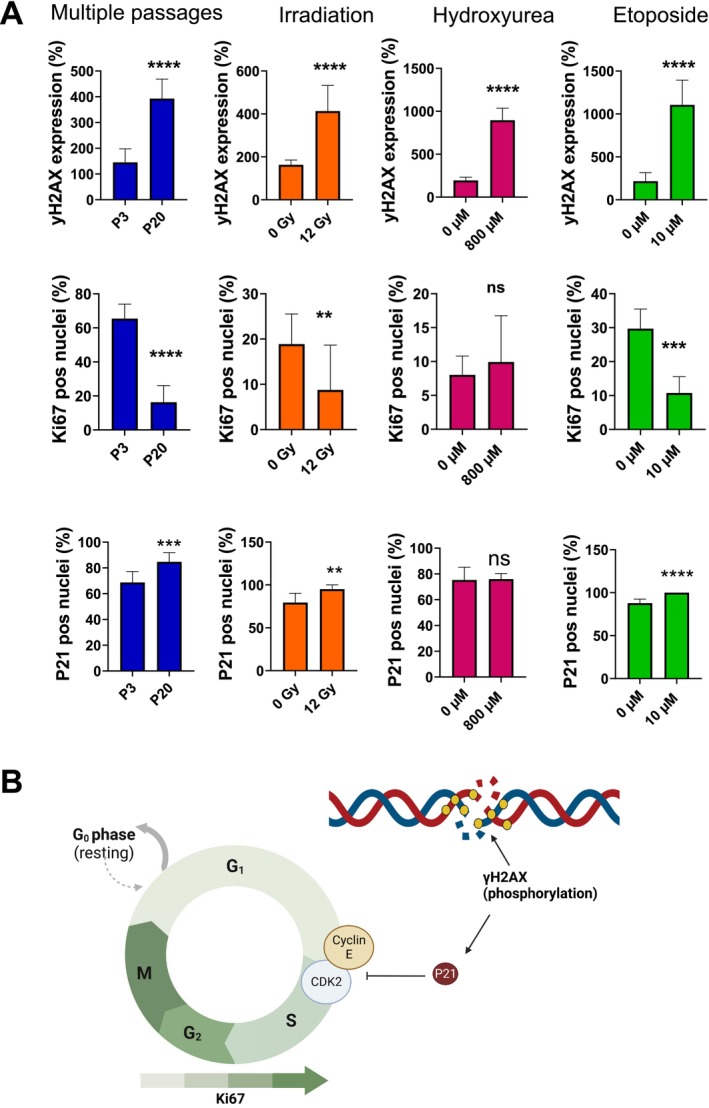
γH2AX foci, Ki‐67, and p21‐positive nuclei immunodetection in senescence‐induced normal human fibroblasts. (A) Representative images of cells at passage 20 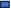, 12 Gy irradiation 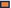, 800 μM hydroxyurea 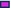, and 10 μM etoposide 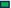 stained with (A) γH2AX staining (green), Ki‐67 (red), and DAPI (blue). Corresponding γH2AX foci count and Ki‐67 expression levels representative of positive nuclei count. p21 staining (blue to red) and DAPI (gray) with p21 levels corresponding to positive nuclei count. (B) Molecular mechanism of action of γH2AX, Ki‐67, and p21 in the response to DNA damage and arrest of the cell cycle activity. Nine repeats from three independent replicates with on averagev> 50 cells per sample. *p* values determined using a *t*‐test and represented as: 0.005 = **, 0.0005 = ***, ≤ 0.00005 = ****, and nonsignificant = ns.

To further confirm understanding of the intracellular biomolecular mechanisms of senescence for each model, molecular markers were analyzed for cells at passage 20, 12 Gy irradiation, 800 μM hydroxyurea, and 10 μM etoposide. Specifically, the phosphorylated form of histone H2 (γH2AX) and KI‐67 were employed to study the extent of DNA damage and replication index (Figure 2), while p21 was utilized to evaluate cell cycle alterations relative to reduced proliferation for all the tested conditions (Figure S5). Based on our results, γHA2X foci levels increased in HFF cells under all senescence induction conditions. Cells at passage 20 and irradiated at 12 Gy had a similar percentage of γH2AX foci per cell (~300% increase). While the drug‐treated cells (800 and 10 μM of hydroxyurea and etoposide, respectively) had > 500% increase i nγH2AX foci per cell. From the evaluation of Ki67‐positive nuclei, we observed that the percentage of positive cells at passage 20, 12 Gy irradiation, and 10 μM of etoposide significantly decreased compared to their relative control. Only hydroxyurea‐treated cells presented a ~20% increase in Ki67‐positive cells compared to the control; however, this value was not significant. Again, these results provide evidence that significant replicative differences are present between different senescence induction models.

Analysis of the expression of p21 positive nuclei in high‐passaged and chemically treated cells indicated an approximate 10%–20% increase of p21‐positive cells, which is significant for cells at passage 20, 12 Gy irradiation, and 10 μM etoposide compared to the matched control. While for hydroxyurea‐treated cells the percentage of p21‐positive cells was similar between treated and control samples, the difference was not significant. Considering the general mechanism for double‐stranded DNA damage (γH2AX) and cell cycle activity (Ki67 and P21) in the induction of senescence, our results show that the enhanced expression of γH2AX foci is associated with alterations in the cell cycle phase and proliferation rate for high passaged cells (P20) and cells treated with irradiation (12 Gy) and etoposide (10 μM), but not for hydroxyurea‐treated cells.

### 3.4 Metabolic Profiles of the Senescence‐Induced Phenotypes

**FIGURE 3:**
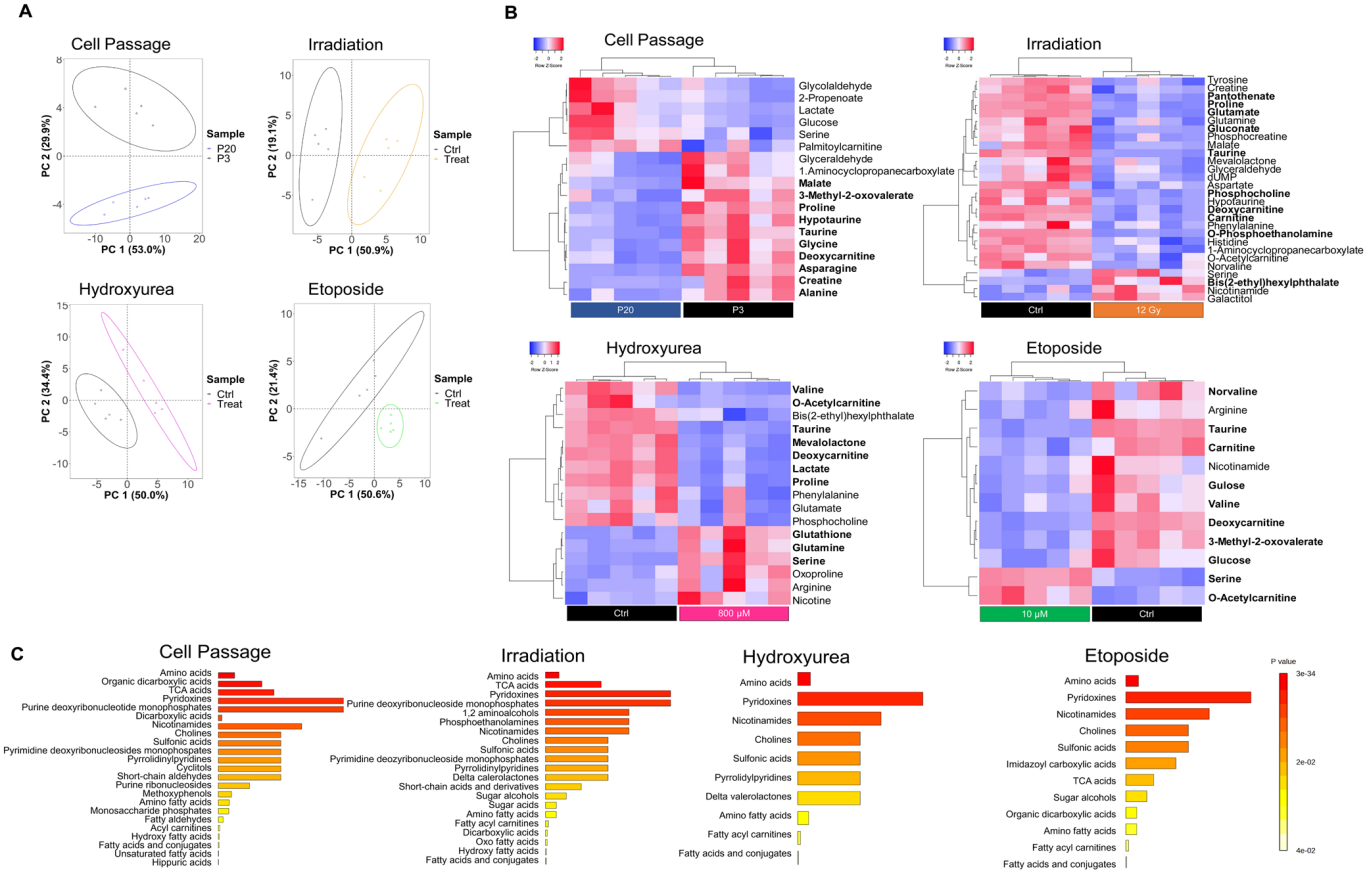
Metabolic pathway dysregulation is heterogeneous across senescence induction methods. (A) PCA pairwise analysis of metabolites altered in the different senescence‐induced cells. For each treatment group, five replicates were used. Data points in the PCA score plot were centered and pareto‐scaled. (B) Heatmap cluster analysis of significant intracellular metabolites (*p* < 0.05) altered upon senescence induction. In bold are the metabolites that contribute to the separation of features based on associated PLS‐DA analyses. Clustering and distance functions are Ward and Euclidean, respectively. Normalized areas indicate chromatographic peak areas that have been normalized based on the QC samples to compensate for batch effects. (C) Enrichment analysis of metabolic classes based on the hypergeometric test. Topological analysis was based on betweenness centrality. *FDR* < 0.05 was deemed significant. Normalized areas indicate chromatographic peak areas that have been normalized based on the QC samples to compensate for batch effects.

To investigate the global metabolic changes of the different senescence models, untargeted metabolomics was performed on HFF‐1 cells in response to all induction methods. Upon assessing the stability of each LCMS run for each condition (Figure S6), a pairwise comparison was performed between treated and nontreated samples to investigate the separation of features, their expression levels, and metabolite classes altered in response to each treatment type (Figure 3). A clear separation of features was detected between control and treated samples across all treatment conditions (Figure 3a). The metabolites contributing to this separation were identified following a partial least squared discriminant analysis (PLS‐DA) analysis (Figure S7) and are listed in Table S10. In passage 20 cells, the most significant metabolites with a log2 fold change > 1.5 were identified as asparagine (−1.80) and citrate (−1.55). In irradiated cells, significantly altered metabolites were identified as gluconate (−2.15) and bis(2‐ethyl)hexylphthalate. In cells treated with hydroxyurea, the metabolites significantly contributing to the separation included o‐acetylcarnitine (−2.79), while in etoposide‐treated cells, the most significantly altered metabolites included 3‐methyl‐2‐oxovalerate (−1.52) (Figure 3b). Amino acids were the most significant class of metabolites altered across all conditions. Serine and taurine were amino acids most significantly dysregulated among all induction methods. Serine levels were upregulated, while taurine levels were downregulated across all treatment conditions. Significantly downregulated levels of deoxycarnitine were also representative of all the tested conditions (Figure 3c). Metabolites were further analyzed in the treatment media of cells following passage 20, 12 Gy irradiation, and treatment with 800 μM hydroxyurea and 10 μM etoposide (Table S11). Among the identified significantly altered metabolites, phenylalanine was downregulated in high passage and etoposide‐treated cells, while its expression was upregulated in irradiated cells and those treated with hydroxyurea. Measured tryptophan levels in collected treatment media were lower for the passaged, irradiated, and etoposide‐treated cells, while tryptophan levels were elevated in response to treatment with hydroxyurea. These results show that overall amino acid metabolism dysregulation is highly conserved across senescence induction methods.

### 3.5 Proteomics Profiles of the Senescence‐Induced Phenotype

**FIGURE 4:**
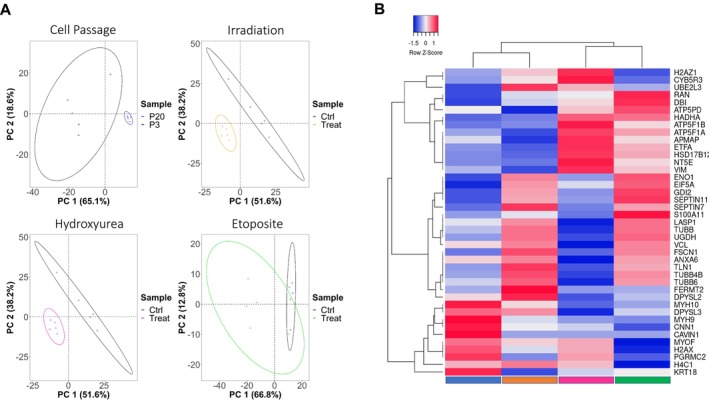
Label‐free proteomics analysis of the protein features identified in HFF‐1 cells. Cells at passage 20 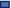, and following 12Gy IR for 1 week 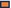, 800 μM hydroxyurea for 2 weeks 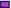, and 10 μM etoposide for 1 week 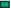. (A) PCA pairwise analysis of proteins altered in the different senescence‐induced cells. For each treatment group, five replicates were used. Data points in the two‐dimensional PCA score plot were centrally scaled. (B) Heatmap cluster analysis of significant intracellular proteins (*p* < 0.05) altered upon induction of senescence. Clustering and distance functions are Ward and Euclidean, respectively. Normalized areas indicate chromatographic peak areas that have been normalized based on the QC samples to compensate for batch effects.

Proteomics analysis was performed on cells exposed to all induction methods and, mirroring the metabolomics analysis, expression levels, and biomolecular classification were investigated (Figure 4). A clear separation was observed between the features measured in the treated versus nontreated samples (control) across all senescence induction methods (Figure 4a). The heatmap indicates the most significantly altered proteins and their log2 fold change, common to all the tested samples (Figure 4b). These included proteins involved in organelle biogenesis and transport (TUBB, TUBB4B, and TUBB6), nuclear envelope reassembly (RAN), signaling by Rho GTPases (CAVIN1, H4C1, PGRMC2, FERMT2, MYH10, γH2AX, and MYH9). The results also showed proteins involved in metabolic processes such as ATP synthesis (ATP5F1A, ATP5F1B, ATP5PD, and ETFA), synthesis of pyruvate (ENO1), glycerophospholipid biosynthesis (HADHA), nucleotide catabolism (NT5E), and cytokine signaling in the immune system (VIM and FSCN1). Significantly expressed proteins for passaged cells, cells treated with irradiation, hydroxyurea, and etoposide are highlighted in Table S12 and were used as input data to perform pathway analysis through the Reactome database (Table S13). Results indicate that common significant pathways among all the conditions were the JAK–STAT pathway, a mediator protein known to attenuate cellular senescence and SASP, and the interleukin 12 signaling pathway—a protein known to be a major component within SASP. No individual pathways were identified for passaged cells. For irradiated cells, proteins were significantly correlated to organelle biogenesis; for hydroxyurea‐treated cells, the most significant pathways were related to cellular response to stress, including heat shock factor 1 (HSF1) mediated response. While for the etoposide‐treated cells, the annotated proteins were significantly representative of NF‐kappa‐B (NF‐kB) and Toll‐like receptor (TLR) signaling pathways.

### 3.6 Multiomics Data Integration and Isotope Tracing of the Identified Metabolic Pathways

To give more insights into the metabolic pathways that were altered upon induction of senescence, a data integration study was performed through joint pathway analysis with MetaboAnalyst software (Table S14) (Debacq‐Chainiaux et al. 2009). Thus, significant proteomics and metabolomics output data (gene ontology, compound name with relative fold change) were combined for each tested condition. The joint pathway analysis enabled the differentiation of pathways significantly enriched (*FDR* < 0.05) in each individual condition. Changes in glycolysis, gluconeogenesis, and pyruvate metabolism were highly representative of both passaged and irradiated cells. Other pathways with a *p* < 0.05 included the pentose phosphate pathway (PPP) for passaged cells, while for irradiated cell changes in phenylalanine, tyrosine, and tryptophan metabolism were present (Table S14). Glutathione and arginine metabolism were significantly (*FDR* < 0.05) enriched in the hydroxyurea‐treated cells, while changes in valine, leucine, and isoleucine metabolism were representative of etoposide cells.

## 4 Discussion

Senescence induction cell models are crucial for studying aging and related diseases. However, there is no standard methodology to decide which induction model to use. Therefore, phenotyping these models is essential because underlying biomolecular changes can significantly impact the accuracy and can lead to misinterpretation of experimental results.

**FIGURE 5:**
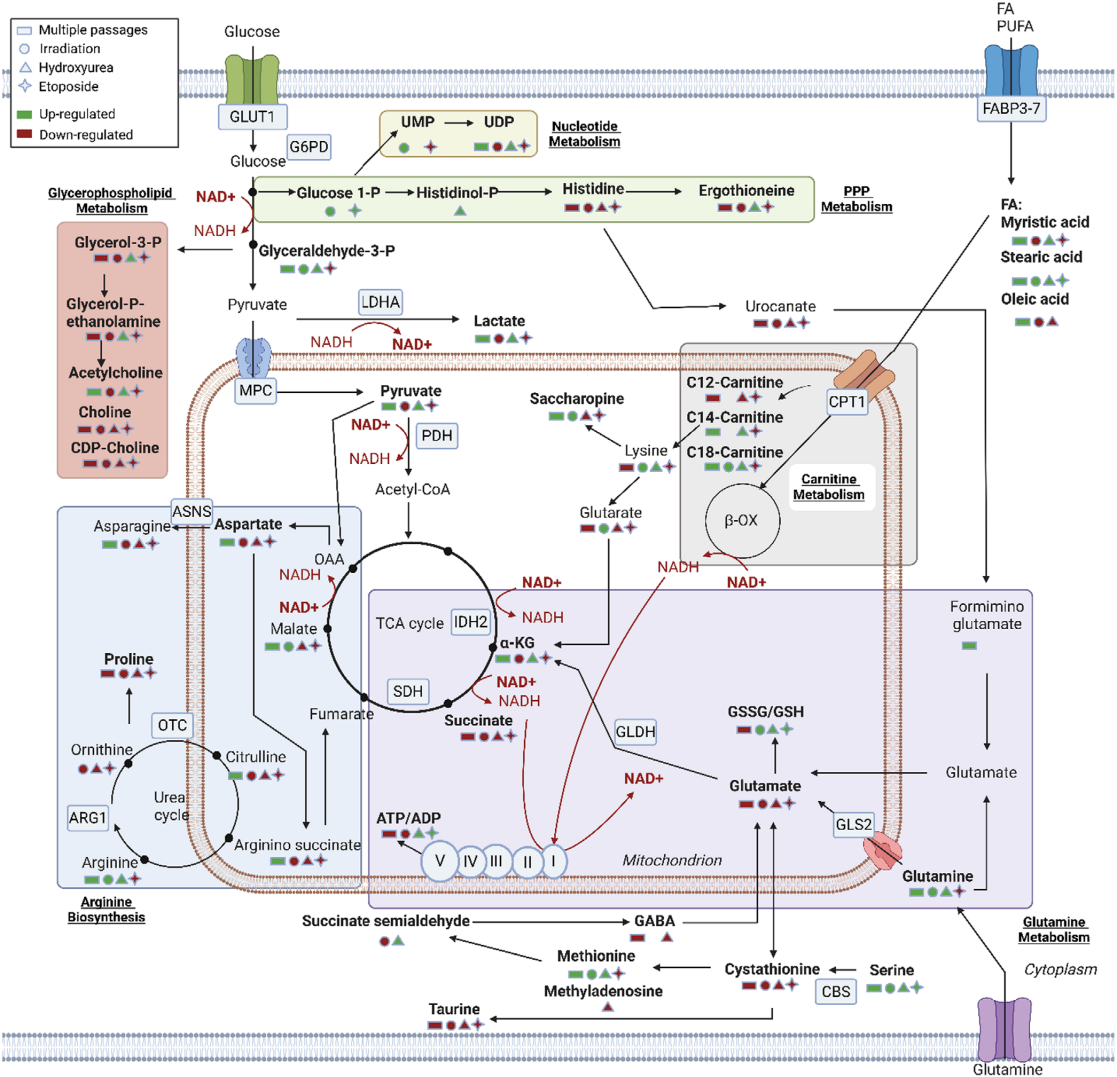
Combined protein and metabolic switching observed across senescence induction methods. These changes varied across senescence models: Replicative senescence showed enriched glutamine metabolism with increased TCA cycle (α‐ketoglutarate) and urea cycle (arginine) metabolites. Irradiation‐induced senescence highlighted glucose‐1‐phosphate and uridine monophosphate, indicating pentose phosphate pathway activation. Hydroxyurea‐induced senescence featured elevated methionine and succinate semialdehyde in glutathione metabolism. All models shared a high oxidized/reduced glutathione ratio. Etoposide‐induced senescence showed depleted pathways but enriched serine, taurine, and saturated fatty acids.

In this study, we reveal consistency in the expression of standard senescence markers commonly used in the biogerontology community, including elevated SA‐β‐Gal expression, p21 levels, and γH2AX DNA damage, across various senescence induction protocols. However, associated metabolic and proteomic profiles were far less consistent (Figure 5). These data show both common and distinct biomolecular profiles across senescence models induced by physical and chemical stressors, identifying a range of novel therapeutic targets for senescent cell clearance.

### 4.1 All Senescence Induction Methods Show Similar Molecular Markers of Senescence Although at Different Magnitudes

SA‐β‐Gal expression is a widely used biomarker of senescence both in vitro and in vivo (Crowe et al. 2014). We found that SA‐β‐Gal expression was elevated across all senescence induction conditions, demonstrating abnormal lysosomal functionality directly related to senescence. In parallel, other senescence markers such as cellular flattened morphology, deformability, DNA damage, cell cycle arrest, and elevated p21 expression confirmed the presence of a senescent phenotype (Wu et al. 2020). All tested conditions were positive for these molecular markers, except for hydroxyurea‐treated cells where no significant changes in morphology or alterations in the cell cycle and p21 expression were detected. Hydroxyurea‐treated cells were the most deformable cells compared to etoposide‐treated, irradiated, and late‐passaged cells. These findings indicate that for hydroxyurea‐treated cells, elevated β‐Gal levels are associated with an alternative senescence‐like subphenotype related to reversible inhibition of ribonucleotide reductase (RR), a critical enzyme that converts ribonucleosides into deoxyribonucleosides, which are required for DNA synthesis and DNA damage repair.

### 4.2 Serine and Taurine Were Common Metabolic Markers of Senescence‐Like Induced Cell Models

LCMS‐based untargeted metabolomics and label‐free proteomic analyses were used to identify specific biomolecular pathways associated with each senescence‐induced model. High levels of serine and low levels of taurine were common metabolic traits of all the tested induction conditions—providing potential new actionable targets for future senescence detection and the development of senolytic therapies. Different pathways via which serine contributes to senescence have been extensively reviewed, which include epigenetic modification (methylation), maintenance of NADPH and GSH levels, and immune regulation (Singh et al. 2023). Metabolomics data from our work confirm these observations. Additionally, taurine is directly associated with senescence and its deficiency has been detected in several age‐related diseases (Johnson et al. 2011). Taurine is metabolized from methionine and cysteine and is involved in many physiological functions such as bile acid conjugation, osmoregulation, antioxidation, and detoxication. Because exposure to radiation can increase ROS, the role of taurine is evidenced as being protective, inhibiting ROS, alongside an enhancement in cysteine and glutathione turnover, again both mechanisms being protective against ROS production (Golla et al. 2017; Locasale 2013).

Cells can acquire serine through degradation from cellular proteins in the lysosome (Singh et al. 2023). However, at the protein level, we detected downregulation of ubiquitinated proteins (Table S12), suggesting impaired protein degradation in our cell models and a consequence of impaired proteostasis, which is another trait of cellular senescence. Serine can also derive from de novo synthesis from glucose and glutamate (Krall et al. 2016), and in the tracing experiments, the carbon serine skeleton was observed to be primarily derived from glucose, while glutamine‐derived glutamate contributed to the nitrogen group of the molecule.

### 4.3 The Induction of Different Damage Stimuli Revealed Metabolic Changes Preferentially Directed Toward a Reductive Metabolism and Altered Expression of Proteins Involved in Several Proinflammatory Pathways

Within the multiple passaged cell models, it was observed that asparagine and citrate metabolites were significantly altered. Asparagine can sustain amino acid homeostasis and maintain the mTOR signaling pathway, therefore providing cells with the nutritional requirements for sustaining cellular proliferation (Deng et al. 2020; Hettmer et al. 2015). Asparagine depletion in tumor cells has been shown to activate p53/p21‐dependent cell cycle arrest and protect cells from apoptosis, which are both hallmarks of senescence (Lamberti et al. 2020). Accordingly, findings show low levels of asparagine associated with high expression of p21 in cells at passage 20 compared to early passaged cells. Additionally, parallel proteomics analysis indicated increased expression of Late endosomal/lysosomal adaptor and MAPK and mTOR activators (LAMTOR), which sense the impaired nutritional state of cells, can signal the lysosome trafficking in cells and regulate innate and adaptive immunity (Bohn et al. 2007; James et al. 2016). Currently, there is little evidence indicating the role of asparagine in cell‐based senescence induction models. Our findings are the first to indicate the critical role asparagine plays in regulating senescence phenotype development in nonsenescent cells and lay the basis for further investigation of its metabolic role and future target potential in senescence. High intracellular citrate levels were also observed and have been found in primary oral fibroblasts together with low levels of TCA cycle metabolites, including malate, whose low levels were also found in our findings (Zhao et al. 2022). The role of citrate in senescence is correlated to excessive lipid biosynthesis (Fan et al. 2013) which is in line with our findings where increased levels of proteins of fatty acid synthesis (HADHA, ECHS1, and HSD17B12) were detected. Stable isotope tracing experiments also indicated citrate in the late passaged cells derives from the reductive metabolism of glutamine. This reductive TCA flux suggests a move from a pro‐ROS to a chemoprotective phenotype (Magalhaes et al. 2023).

In cells treated with ionizing radiation, metabolic dysregulation in the glycogen catabolism (gluconate) and PPP was detected, therefore indicating that changes in the upper pathways of glycolysis display a level of plasticity in cells upon irradiation. The low levels of gluconate detected in radiation‐treated compared to the nontreated cells suggest a reduced activity of the oxidative arm of the PPP pathway and a preference for the reduced activity. The reduced PPP was further confirmed through the ^13^C_6_‐glucose isotope tracing experiment. At the protein level, the downregulated expression of proteins involved in DNA double‐strand break response (H4C1, H2BC12, γH2AX) was detected, indicating the presence of disrupted mechanisms of DNA repair. This was paralleled by the downregulation of RHO GTPases‐related proteins that—upon ionizing radiation—are known to regulate the DDR pathway at the early stages of DNA damage recognition (Dipankar et al. 2021). RHO GTPases have a role in inflammation (King 2004) and cell migration/adhesion, and from our findings, their downregulation was associated with reduced levels of proteins involved in the interleukin‐12 signaling pathways.

Considering cells treated with chemicals, we observed that in the hydroxyurea‐treated cells, high levels of glutathione were detected, which is in line with the activity of hydroxyurea in inducing high levels of nitrogen radicals, therefore producing high levels of ROS (Andrade et al. 2021) Also, high levels of oxoproline were detected in these cells, which further highlighted the importance of reactive oxygen/nitrogen species in these cells. The tracing analysis further confirmed the incorporation of glutamine‐derived carbons in the structure of glutathione. The role of hydroxyurea in inducing high production of ROS in these cells was further confirmed at the protein level, where enhanced regulation of proteins involved in the detoxification of ROS was detected, including P4HB, CYCS, PRDX3/6, and SOD2. While in the etoposide‐treated cells, changes in the metabolite 3‐methyl‐2‐oxovalerate were found to be significantly relevant; levels in the treated samples were lower compared to the nontreated cells. This metabolite is a product of branched‐chain amino acids, which has been related to quiescence, not senescence (Fendt et al. 2013) and might therefore be critical to distinguishing between the two phenotypic states. Similarly to irradiation, also for etoposide, we observed downregulated levels of proteins involved in RHO GTPases signaling pathways and chromatin organization, suggesting a critical role of this drug in these pathways.

### 4.4 Multiomics Data Indicate Glycolytic Switching to a Reductive Phenotype

**FIGURE 6:**
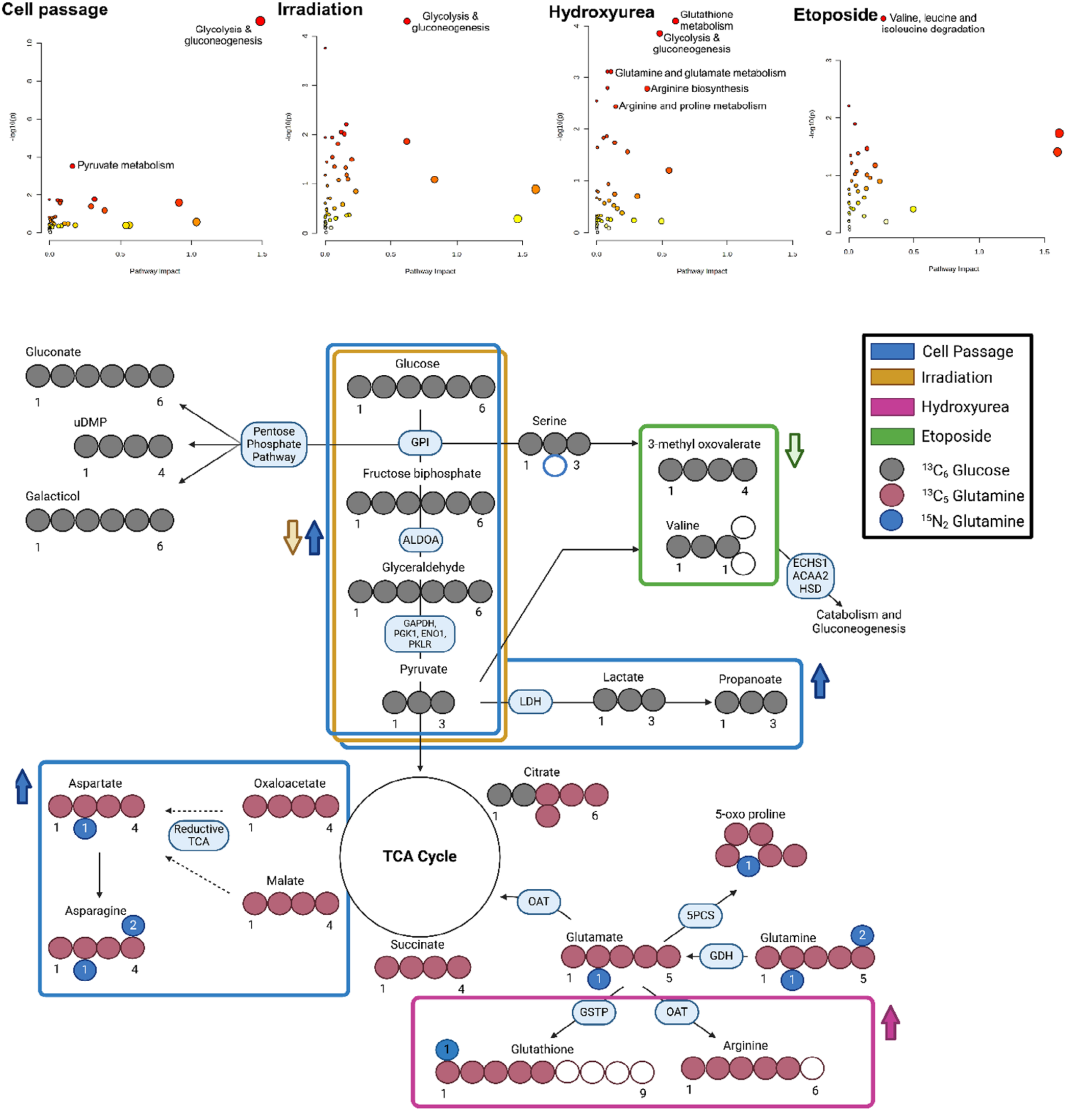
Stable isotope analysis confirms a range of different metabolic switching processes. (A) Pathway enrichment analysis of HFF‐1 cells at passage 20, and following 12Gy IR for 1 week, 800 μM hydroxyurea for 2 weeks, and 10 μM etoposide for 1 week. Enrichment analysis, performed using the freely accessible MetaboAnalyst software (www.metaboanalyst.ca), was based on betweenness centrality. The tight interpretation method was used by combining proteins and metabolites into a single query. A *p* < 0.05 and pathway impact > 0.1 were deemed significant. (B) Manually created schematic, derived from Compound Discoverer isotope ratio data, showing how [^13^C_6_]‐glucose and [^13^C_5_, ^15^N_2_]‐glutamine can be metabolized through reductive and/or oxidative pathways.

Joint pathway analysis (Figure 6a and Table S14) indicates a range of pathways significantly enriched (*FDR* < 0.05) by each individual induction method. Changes in glycolysis, gluconeogenesis, and pyruvate metabolism were representative of both passaged and irradiated cells. Other pathways with a nonadjusted *p* < 0.05 included PPP for passaged cells, while for irradiated cells we observed relevant changes also in phenylalanine, tyrosine, and tryptophan metabolism (Table S14). Glutathione and arginine metabolism were significantly (*FDR* < 0.05) enriched in the hydroxyurea‐treated cells, while changes in valine, leucine, and isoleucine metabolism were representative of etoposide cells.

To gain insights into the metabolic changes involved in the development of senescence, we examined the incorporation of carbon and nitrogen groups from the [^13^C_6_]‐glucose and [^13^C_5_, ^15^N_2_]‐glutamine tracers into the downstream metabolites (Figure 6b). We propose that upon induction of senescence, glucose and glutamine metabolism are directed toward anabolic (reductive) or catabolic (oxidative) metabolic processes depending on the method of induction. From the results, we observed for passaged cells a decrease in ^13^C‐glucose labeled carbons to form citrate (m + 2) and an increase in the ^13^C‐glutamine carbons (m + 4), which is indicative of the reductive reactions of glutamine utilization in the TCA cycle (Fendt et al. 2013). Decrease in m + 2 of carbon—but not m + 4—incorporation into citrate was observed also for irradiated and hydroxyurea‐treated cells, while cells treated with etoposide did not present this rewiring of carbon incorporation into citrate.

Deoxyuridine‐monophosphate (dUMP), gluconate, and galactitol are products of the PPP pathway where glucose undergoes a series of reductive reactions (Stincone et al. 2015) For these compounds, entry of ^13^C‐glucose carbons (m + 6) was detected to increase in passaged and etoposide‐treated cells but not for the irradiated and hydroxyurea‐treated cells. We also detected incorporation of ^15^N‐glutamine nitrogen groups into amino acids (e.g., glutamate, aspartate, asparagine, and valine) and glutathione (Figure 6b, Figures S8–S11). Enhanced entry of ^15^N‐glutamine nitrogen into glutamate was detected for passaged cells, irradiated, and etoposide‐treated cells, but not for etoposide. A similar trend was observed for ^15^N‐glutamine nitrogen incorporation into aspartate. Interestingly, in cells at passage 20, ^15^N‐glutamine nitrogen entry into asparagine was considerably decreased for m + 2 compared to cells at passage 3 (Figures S6–S9). Regarding glutathione, a significant decrease in ^15^N‐glutamine was observed for m + 2 nitrogen in all the tested conditions, and no incorporation was detected for the drug‐treated cells (hydroxyurea and etoposide).

Previous studies that have focused on metabolic phenotyping of endothelial senescent cells have also indicated significant redox alterations and associated changes in amino acid metabolism. Yi et al. (2020) conducted an NMR‐based metabolomic analysis on human umbilical vein endothelial cells (HUVECs) during replicative senescence. Their findings indicated that senescent cells exhibited changes in amino acid metabolism, including increased levels of glutamine, aspartate, and asparagine at early stages, followed by a decrease at later stages. These metabolic alterations are closely associated with oxidative stress, impaired energy metabolism, and blocked protein synthesis—and mirror results we have also observed. Sabbatinelli et al. (2019) also reviewed this area and highlighted endothelial senescent cells shifting metabolism toward a more glycolytic state despite high oxygen levels. These metabolic alterations are known to drive the production and secretion of various SASP factors, including cytokines, chemokines, and proteases, which contribute to chronic inflammation and tissue remodeling—all molecular features that translate to the observed pathophysiology of aging tissue phenotypes.

## 5 Conclusion

This research demonstrates that the integration of molecular biology, rheo‐morphology, metabolomics, and proteomics analysis delineates precise biomolecular profiles of different senescence‐induced phenotypes. These phenotypes display a range of different intracellular biomolecular profiles and demonstrate that the method of induction significantly influences cell metabolic, proteomic, and rheo‐morphological profiles. Methods of senescence induction are not interchangeable, and careful consideration needs to be made when planning out future in vitro experimentation or when comparing across different methodologies. There was consistent metabolic dysregulation observed across all induction methods. Moreover, our data shows that upon induction of a senescence‐like phenotype, cells rewire their metabolism toward reductive rather than oxidative pathways. This metabolic shift highlights the association between senescence and reductive metabolism, suggesting that senescent cells favor pathways that minimize oxidative stress.

A caveat associated with our approach does arise from the use of the HFF‐1 cell line and the need to develop an understanding of not only how different senescence induction methods alter the underlying metabolic phenotype, but also how conserved the results are across cell lines originating from different tissues. With estimates of over 200 different functional cell types in an adult human body (Weeden et al. 2023), the biogerontology community needs to be aware of how conserved underlying metabolic changes caused by senescence induction are. Plans to document observed metabolite differences in different senescence cell/induction experiments open the opportunity for further investigations to target metabolic redox circuits as antiaging/senolytic/senomorphic therapies and in the treatment of age‐related diseases via drug and fragment screening approaches. By understanding and manipulating these metabolic pathways, interventions can be developed that can mitigate the detrimental effects of cellular senescence, thereby improving health span and treating age‐related conditions.

## Author Contributions

Domenica Berardi performed most of the experiments and wrote the manuscript; Gillian Farrell, Abdullah Alsultan, Ashley McCulloch, Nicole M. Hall, and Rui Pedro Pereira Sousa contributed to the experiments; Melanie Jimenez, Colin Selman, Ilaria Bellantuono, Caroline H. Johnson, and Zahra Rattray designed experiments and edited the manuscript. Nicholas J.W. Rattray led the research. All authors have read and approved the final manuscript.

## Conflicts of Interest

The authors declare no conflicts of interest.

## Supporting information


Data S1.

## Data Availability

All metabolomics, proteomics and tracer experimental data will be available through MetaboLights and ProteomeXchange respectively. We actively host all our data on there and at the time of submission we are going throught the process of data upload. Please contact the lead author for links to data access.
