# Supplementary material for: Senescence Cell Induction Methods Display Diverse Metabolic Reprogramming and Reveal an Underpinning Serine/Taurine Reductive Metabolic Phenotype
Source: Aging Cell. 2025 Jun 18;24(8):e70127. doi: 10.1111/acel.70127 (PMC12341781; doi:10.1111/acel.70127)
Supplement: Supplementary file 1 — Data S1. [file ACEL-24-e70127-s001.docx]

SUPPLEMENTARY

Senescence cell induction methods display diverse metabolic reprogramming and reveal an underpinning serine/taurine **reductive metabolic** phenotype.

Domenica Berardi^1,2^ | Gillian Farrell^2^ | Abdullah AlSultan^2,3^ | Ashley McCulloch^4^ | Nicole M. Hall^4,5^ | Rui Pedro Pereira Sousa^4,5^ | Melanie Jimenez^4,5^ | Colin Selman^6^ | Ilaria Bellantuono^7^ | Caroline H. Johnson^1^ | Zahra Rattray^2^ | Nicholas J. W. Rattray^2,8^

^1^ Department of Environmental Health Sciences, Yale School of Public Health, Yale University, New Haven, Connecticut, USA

^2^ Strathclyde Institute of Pharmacy and Biomedical Science, University of Strathclyde, Glasgow, UK

^3^ Faculty of Pharmacy, Kuwait University, Safat, Kuwait

^4^ Wolfson Wohl Cancer Research Centre, School of Cancer Sciences, University of Glasgow, Glasgow, UK

^5^ Department of Biomedical Engineering, University of Strathclyde, Glasgow, UK

^6^ School of Molecular Biosciences, University of Glasgow, Glasgow, UK

^7^ Department of Oncology and Metabolism, Healthy Lifespan Institute and MRC–Arthritis Research UK Centre for Integrated Research Into Musculoskeletal Ageing, University of Sheffield, Sheffield, UK

^8^ Strathclyde Centre for Molecular Bioscience, University of Strathclyde, Glasgow, UK

Correspondence:

Nicholas J. W. Rattray ([nicholas.rattray@strath.ac.uk](mailto:nicholas.rattray@strath.ac.uk))

***Supplementary Table 1|*** *Elution gradient for the chromatographic separation of metabolites.*

| Time (min) | Flow [ml/min] | % B | Curve |
| --- | --- | --- | --- |
| 0.00 | 0.300 | 20.0 | 5 |
| 11.00 | 0.300 | 60.0 | 5 |
| 15.00 | 0.300 | 20.0 | 5 |
| 20.00 | 0.300 | 20.0 | 5 |

***Supplementary Table 2|*** *LC-MS/MS settings for metabolomics.*

| **Global parameters** |  |
| --- | --- |
| Expected LC peak width | 20 |
| Default charge state | 1 |
| Internal mass calibration | EASY-IC™ |
| **Ion source properties** |  |
| Ion source type | H-ESI |
| Spray voltage | Static |
| Positive ion (V) | 3900 |
| Negative ion (V) | 2500 |
| Gas mode (Arb) | 40 |
| Sheath Gas (Arb) | 10 |
| Sweep Gas (Arb) | 1 |
| Ion transfer tube temp (℃) | 320 |
| Vaporizer temp (℃) | 300 |
| **Scan parameters** |  |
| Full scan |  |
| Orbitrap resolution | 120,000 |
| RF Lens (%) | 70 |
| Polarity | Pos and neg |
| Intensity threshold | 1.0E4 |
| Precursor fit |  |
| Fit threshold | 70% |
| Fit window | 0.7 m/z |
| Charge state | 1-2 |
| Targeted mass |  |
| Mass tolerance | 5 ppm (low and high) |
| Collision energy per compound | True |
| Targeted mass difference |  |
| Mass tolerance | 10 ppm (low and high) |
| Apex window | 50% |
| MS/MS |  |
| Isolation window | 0.7 m/z |
| Orbitrap resolution | 30,000 |

**Supplementary Table 3|** Compound Discoverer settings for the workflow “Stable Isotope Labeling with ID using Local Databases”.

| 1. Input files |  |
| --- | --- |
| 2. Select spectra | Any polarity |
| 3. Align retention times | Adaptive curve. 5 ppm mass tolerance |
| 4. Detect compounds | Intensity tolerance is 30%  Mass tolerance is 5 ppm |
| 5. Group compounds | Mass tolerance is 5 ppm  RT tolerance is 0.5 min |
| **Labelled analysis** |  |
| 6. Assign compound annotations | Mass tolerance is 5 ppm  Data source is local mass list and library |
| 7. Analyze labeled compounds | Labeled element is [13]C and [15]N  Max exchange is 25 |
| 8. Mark background compounds |  |
| 9. Search mass list | Local mass list. Mass tolerance of 5 ppm. |
| 10. Predict composition | Mass tolerance is 5 ppm |
| 11. Search local library | Match any ion activation energy  Precursor mass tolerance is 10 ppm  Search algorithm is HighChem HighRes  RT tolerance is 2 min |
| **Untargeted Analysis** |  |
| 6. Fill gaps | Mass tolerance is 5 ppm  S/N is 1.5 |
| 7. Apply QC correction | Min coverage is 50  Max area is 30  Max corrected area (RSD) is 25 |
| 8. Mark background compounds |  |
| 9. Normalize areas | Normalization by constant media |
| 10. Predict composition | Mass tolerance is 5 ppm |
| 11. Assign compound annotation | Mass tolerance is 5 ppm  Data source is local mass list and library |
| 12. Search mass list | Local mass list. Mass tolerance of 5 ppm. |
| 13. Search local library | Match any ion activation energy  Precursor mass tolerance is 10 ppm  Search algorithm is HighChem HighRes  RT tolerance is 2 min |

**Supplementary Table 4|** Elution gradient for proteomics.

| Time (min) | Flow [ml/min] | % B | Curve |
| --- | --- | --- | --- |
| 0.00 | 0.050 | 3.0 | 5 |
| 65.00 | 0.050 | 20.0 | 5 |
| 70.00 | 0.050 | 40.0 | 5 |
| 74.00 | 0.050 | 95.0 | 5 |
| 79.00 | 0.050 | 95.0 | 5 |
| 84.00 | 0.050 | 3.0 | 5 |
| 100.00 | 0.050 | 3.0 | 5 |

**Supplementary Table 5|** LC-MS/MS settings for proteomics.

| **Global parameters** |  |
| --- | --- |
| Expected LC peak width | 10 |
| Default charge state | 2 |
| Internal mass calibration | EASY-IC™ |
| **Ion source properties** |  |
| Ion source type | H-ESI |
| Spray voltage | Static |
| Positive ion (V) | 3400 |
| Negative ion (V) | 3000 |
| Gas mode (Arb) | 25 |
| Sheath Gas (Arb) | 5 |
| Sweep Gas (Arb) | 0 |
| Ion transfer tube temp (℃) | 320 |
| Vaporizer temp (℃) | 75 |
| **Scan parameters** |  |
| Full scan |  |
| Orbitrap resolution | 120,000 |
| RF Lens (%) | 70 |
| Polarity | Pos |
| Intensity threshold | 1.0E4 |
| Monoisotopic peak determination | Peptide |
| Charge state | 2-5 |
| MSMS |  |
| Isolation window | 1,2 m/z |
| Orbitrap resolution | 15,000 |

**Supplementary Table 6** Settings for proteome discoverer

| **Processing workflow** |  |
| --- | --- |
| **Spectrum files RC** |  |
| Protein database | Homo Sapiens (TaxID:9606) |
| Enzyme name | Trypsin |
| Precursor mass tolerance | 20 ppm |
| Fragment mass tolerance | 0.5 Da |
| Regression model | Non-linear regression |
| Parameter tuning | Coarse |
| **Minora** |  |
| Min. trace length | 5 |
| S/N threshold | 1 |
| Max ∆RT (min) | 0.2 |
| PMS confidence at least | High |
| **Spectrum selector** |  |
| Precursor selection | Use MS1 precursor |
| Min precursor mass | 350 Da |
| Max precursor mass | 6500 Da |
| Min peak count | 1 |
| Scan type | Is full |
| Polarity mode | Any |
| **Sequest HT** |  |
| Max missed cleavage | 2 |
| Min peptide length | 6 |
| Max peptide length | 144 |
| **Percolator** |  |
| Target/Decoy selection | Concatenated |
| Validation based on | q-Value |
| Target FDR (Strict) | 0.01 |
| Targeted FDR (Relaxed) | 0.05 |
| **Consensus workflow** |  |
| **MSF Files** |  |
| Merge mode | Globally by search engine type |
| Reported FASTA tale lines | Best match |
| **Feature mapper** |  |
| Perform RT alignment | TRUE |
| Max RT shift (min) | 10 |
| Min S/N threshold | 5 |
| **Precursor ions quantifier** |  |
| Peptide to use | Unique + Razor |
| Consider protein groups | TRUE |
| Precursor abundance based on | Intensity |
| Min replicate features (%) | 60 |
| Protein abundance calculation | Summed abundances |
| Protein ratio calculation | Protein abundance based |
| Max allowed fold change | 100 |
| Hypothesis test | ANOVA (Individual proteins) |
| **PMS Grouper** |  |
| Site probability threshold | 75 |
| **Peptide validator** |  |
| Validation mode | Automatic |
| Target FDR (strict) | 0.01 |
| Target FDR (relaxed) | 0.05 |
| **Peptide and protein filter** |  |
| Peptide confidence at least | High |
| Min peptide length | 6 |
| Min N of peptide sequences | 1 |
| **Protein marker** |  |
| Contaminant database | PD_Contaminants_2015_5fasta |
| Additional marker database | Mus musculus (TaxID:10090) |
| **Protein annotation** |  |
| Aspect | Biological process, Cellular component, molecular function |
| **Protein grouping** | TRUE |
| **Peptide in protein annotation** |  |
| Protein modifications reported | Only for master proteins |
| Modification sites reported | All and specific |


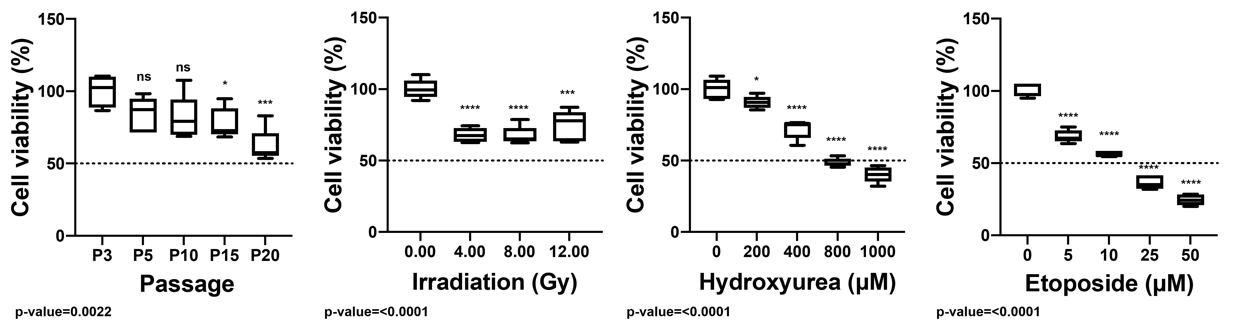


**Supplementary Figure 1|** **Viability response of HFF-1 cells at different passages, different doses of X-Ray, hydroxyurea and etoposide.** Passaged, irradiated and etoposide-treated cells were kept in culture for 1 week, while hydroxyurea cells were treated for 2 weeks. After the incubation time, cells were stained with crystal violet, subsequently solubilised in ethanol, and absorbance was read at 600 nm. p-values have been determined through ANOVA test. Dunnett’s multiple comparison test was used as a follow up to ANOVA test and the p-values were represented as: non-significant=ns, 0.05=*, 0.005=**, 0.0005=***, 0.00005=****.


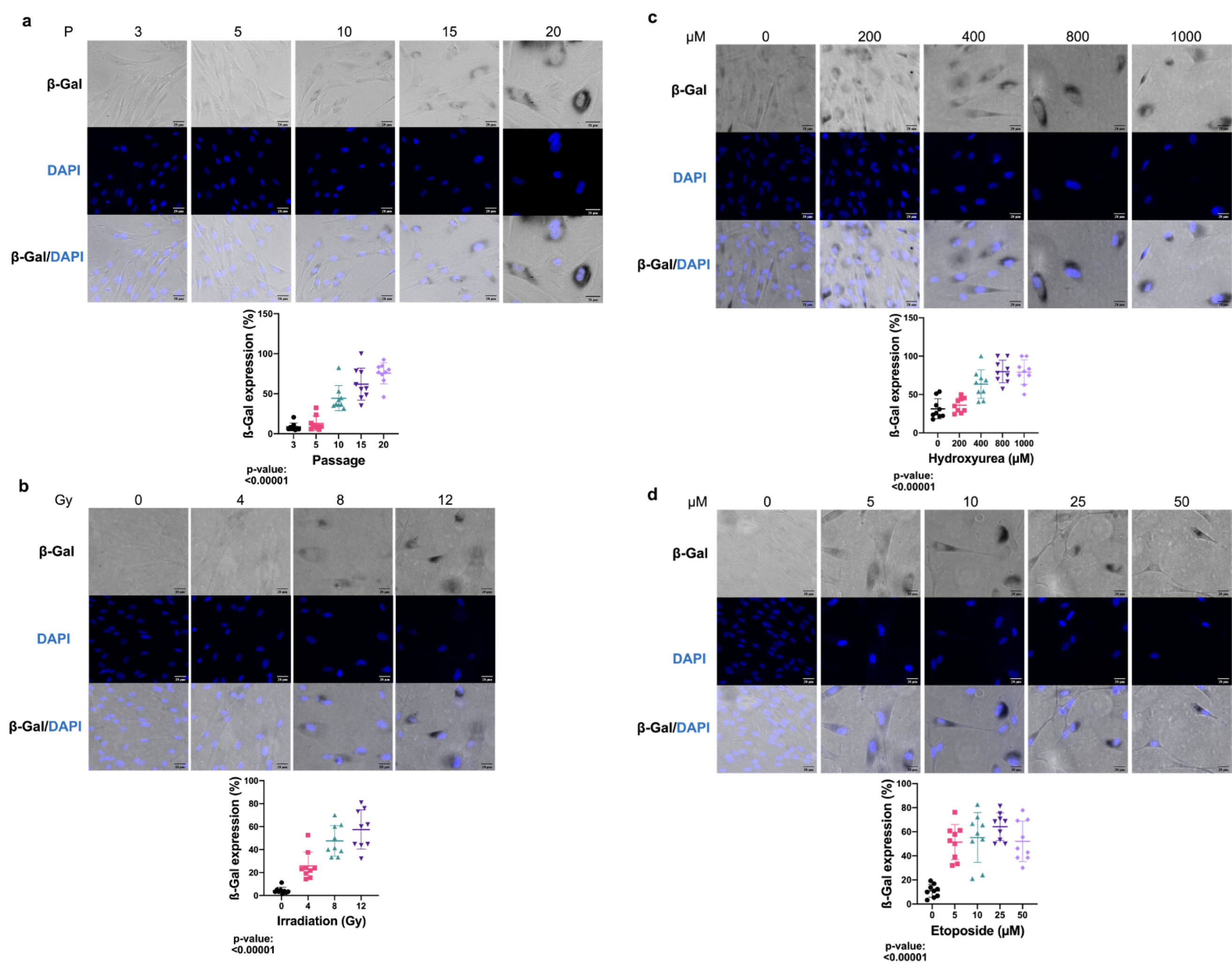


**Supplementary Figure 2|** **The expression of SA-β-Gal in senescence-induced HFF-1 cells.** Representative phase contrast images of SA-β-Gal staining (grey), DAPI immunolabelled nuclei (blue) and composite (β-Gal (grey) and DAPI (blue)) in cells at different passages (a), increasing irradiation doses for 1 week (b), treatment with increasing concentrations of hydroxyurea for 2 weeks (c) and etoposide for 1 week (d). For each condition, corresponding β-Gal expression levels expressed have been reported as a percentage of manually counted stained cells relative to the number of counted nuclei using ImageJ. 9 repeats with on average >50 cells per each sample. p-values have been determined ANOVA test.


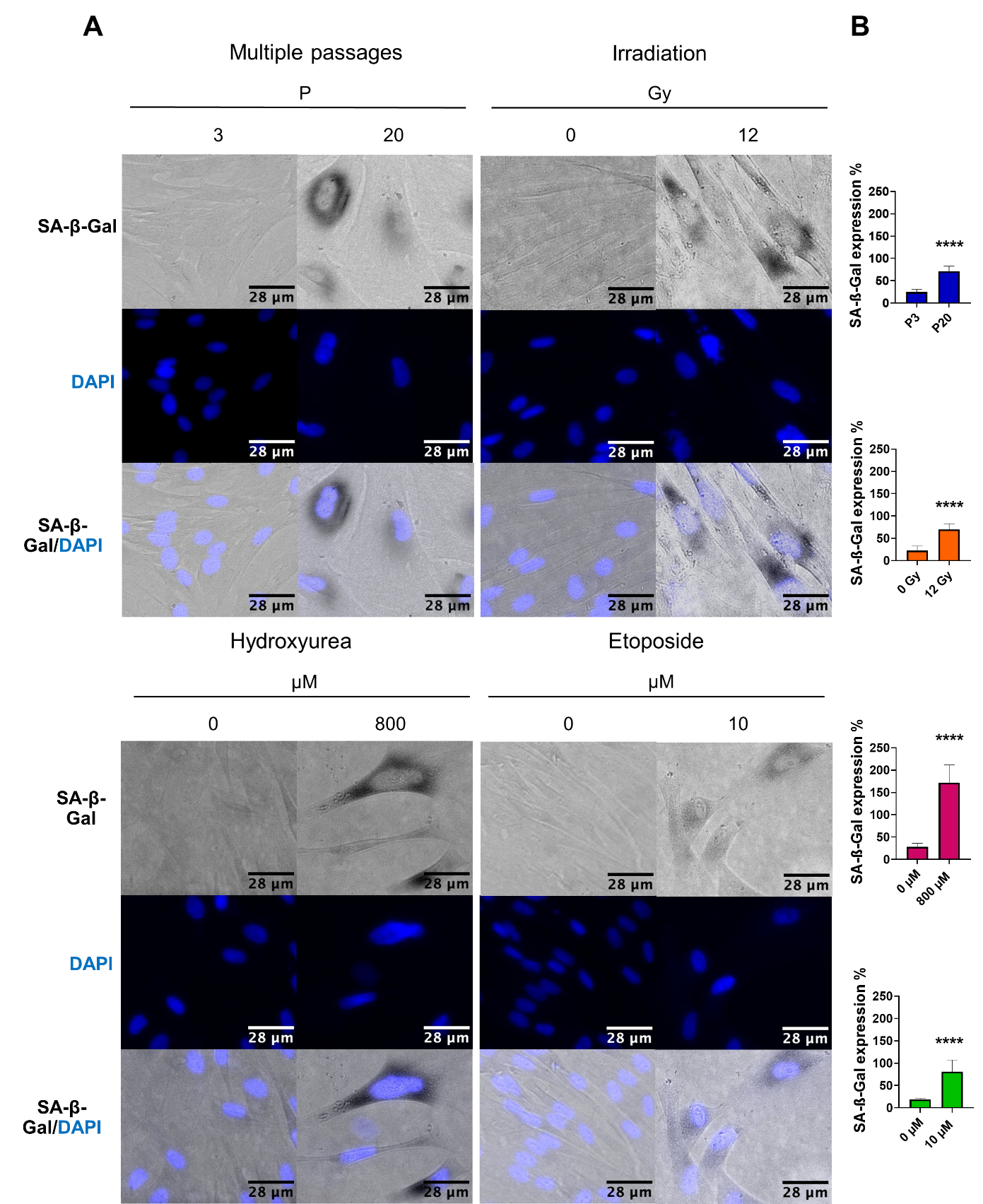


***Supplementary Figure 3|*** ***SA-ß-Gal expression in senescence-induced human fibroblasts****. Representative phase contrast images of SA-ß-Gal staining (grey), DAPI (blue) and composite (SA-ß-Gal (grey) and DAPI (blue)), in cells at passage 3 and 20 , one week post radiation at 12 Gy , 800 µM hydroxyurea for two weeks and 10 µM etoposide for one week (A). Corresponding SA-ß-Gal levels expressed as a percentage of SA-ß-Gal staining intensity relative to the number of nuclei (B), and measured through Cell Reporter. 9 repeats from 3 independent replicates with on average >50 cells per each sample. P-values have been determined through t-test and represented as ≤0.00005=****.*


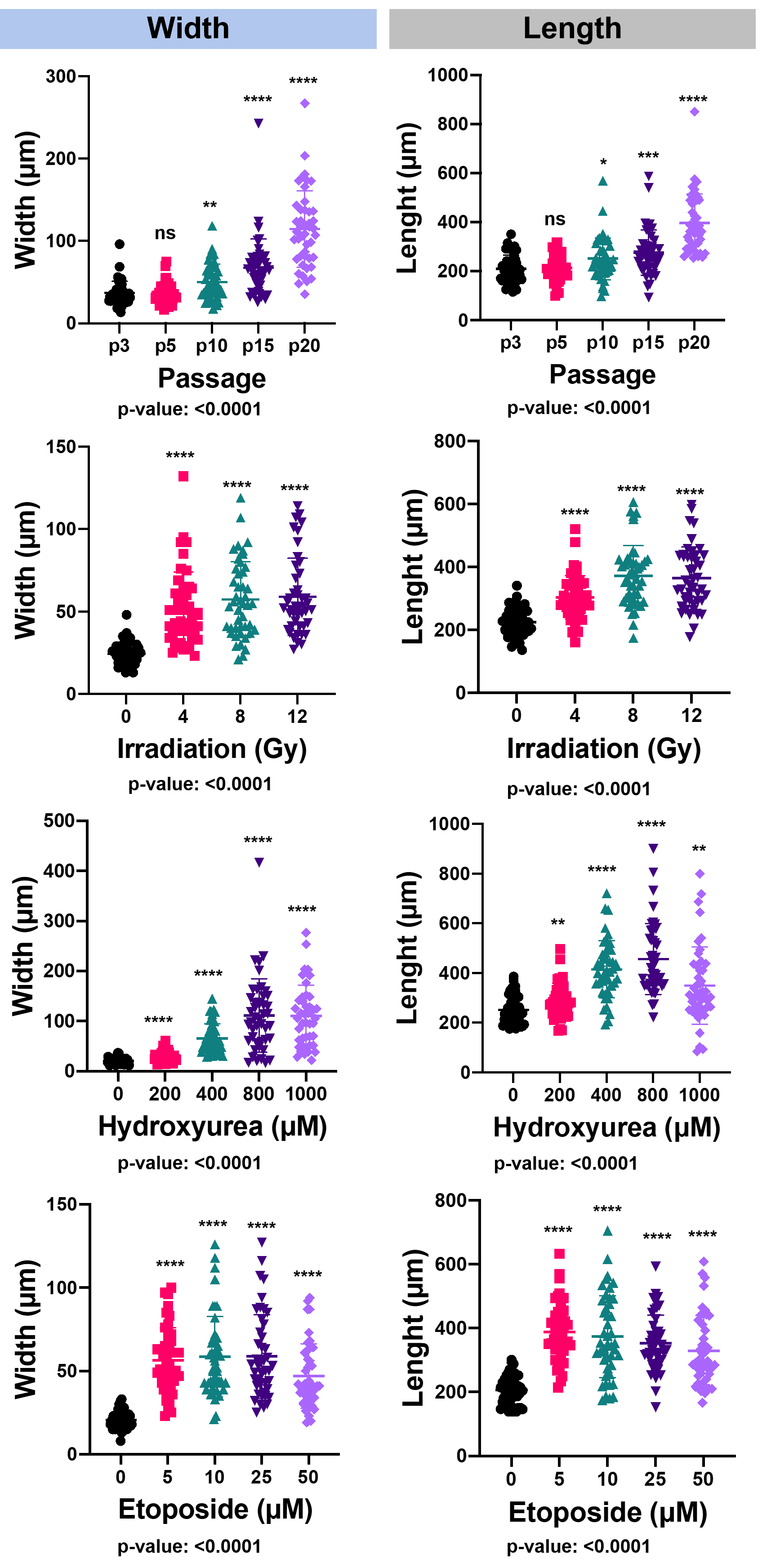


**Supplementary Figure 4|** **Measurements of width and length of HFF-1 cells**. Cells at late passage (P20), 12 Gy irradiation, for 1 week, 800 µM hydroxyurea for 2 weeks, and 10 µM etoposide for 1 week. Cells stained for β-Gal expression have been analysed to measure width and length of individual cells. A total number of 45 cells were analysed per each passage and dosage. Width and length were measured through the line tool in ImageJ and expressed as µm. Dunnett’s multiple comparison test was used as a follow up to ANOVA and corresponding p-values were represented as: non-significant=ns, 0.05=*, 0.005=**, 0.0005=***, 0.00005=****.

**Supplementary Table 7|** Mean area measurements of HFF-1 cells after passage 20, 12 Gy irradiation, and treatment with 800 µM hydroxyurea and 10 µM etoposide through real-time deformability cytometry in the reservoir channel.

|  | A = Mean area in reservoir control (µm^2^) | B = Mean area in reservoir test (µm^2^) | *B - A* |
| --- | --- | --- | --- |
| Passage number | 227.3 ± 84.7 | 347.7 ± 114.8 | ***120.4*** |
| Irradiated cells | 232.3 ± 89.7 | 280.2 ± 121.0 | *47.9* |
| ETP | 265.8 ± 108.0 | 337.4 ± 160.5 | *76.1* |
| HU | 251.2 ± 102.9 | 265.9 ± 130.7 | ***14.7*** |

**Supplementary Table 8|** Mean deformability measurements of HFF-1 cells after passage 20, 12 Gy irradiation, and treatment with 800 µM hydroxyurea and 10 µM etoposide through real-time deformability cytometry in the deformability channel.

|  | A = Mean deformability in channel control (-) | B = Mean deformability in channel test (-) | *B – A* |
| --- | --- | --- | --- |
| Passage number | 0.051 ± 0.025 | 0.053 ± 0.023 | *0.002* |
| Irradiated cells | 0.064 ± 0.032 | 0.059 ± 0.029 | *-0.005* |
| ETP | 0.075 ± 0.028 | 0.072 ± 0.034 | *-0.003* |
| HU | 0.084 ± 0.035 | 0.077 ± 0.040 | *-0.007* |

**Supplementary Table 9|** Mean deformability measurements of HFF-1 cells after passage 20, 12 Gy irradiation, and treatment with 800 µM hydroxyurea and 10 µM etoposide through real-time deformability cytometry in the reservoir channel.

|  | A = Mean deformability in reservoir control (-) | B = Mean deformability in reservoir test (-) | *B - A* |
| --- | --- | --- | --- |
| Passage number | 0.022 ± 0.018 | 0.021 ± 0.017 | ***-0.001*** |
| Irradiated cells | 0.025 ± 0.022 | 0.024 ± 0.020 | ***-0.001*** |
| ETP | 0.030 ± 0.023 | 0.039 ± 0.032 | *0.009* |
| HU | 0.033 ± 0.030 | 0.059 ± 0.045 | ***0.026*** |


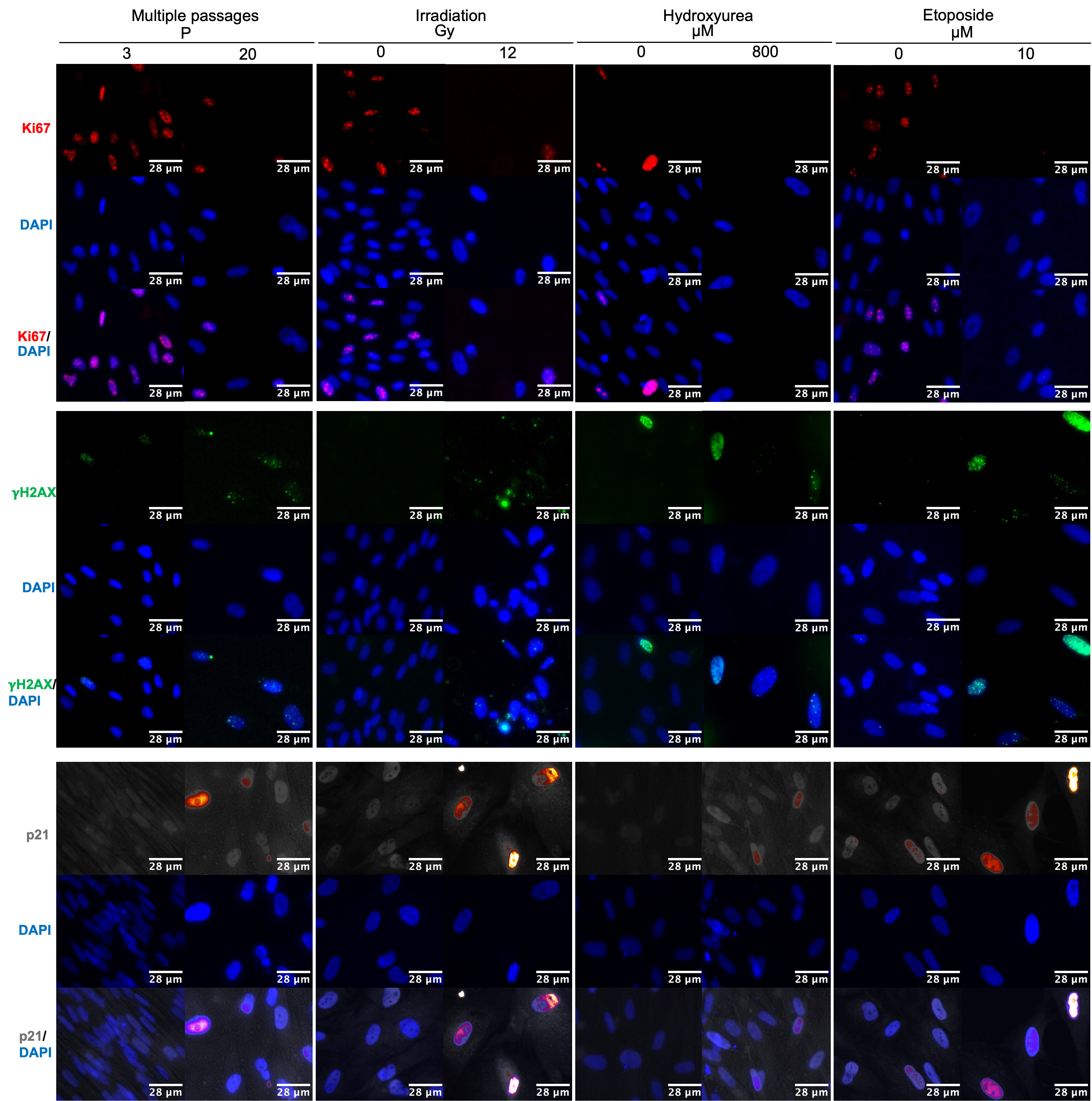


**Supplementary Figure 5|** **ɣH2AX foci, Ki-67 and p21 positive nuclei immunodetection in senescence-induced normal human fibroblasts.** Representative images of cells at passage 20, 12 Gy irradiation, 800 µM hydroxyurea and 10 µM etoposide stained with ɣH2AX staining (green), Ki-67 (red), p21 (grey) and DAPI (blue).


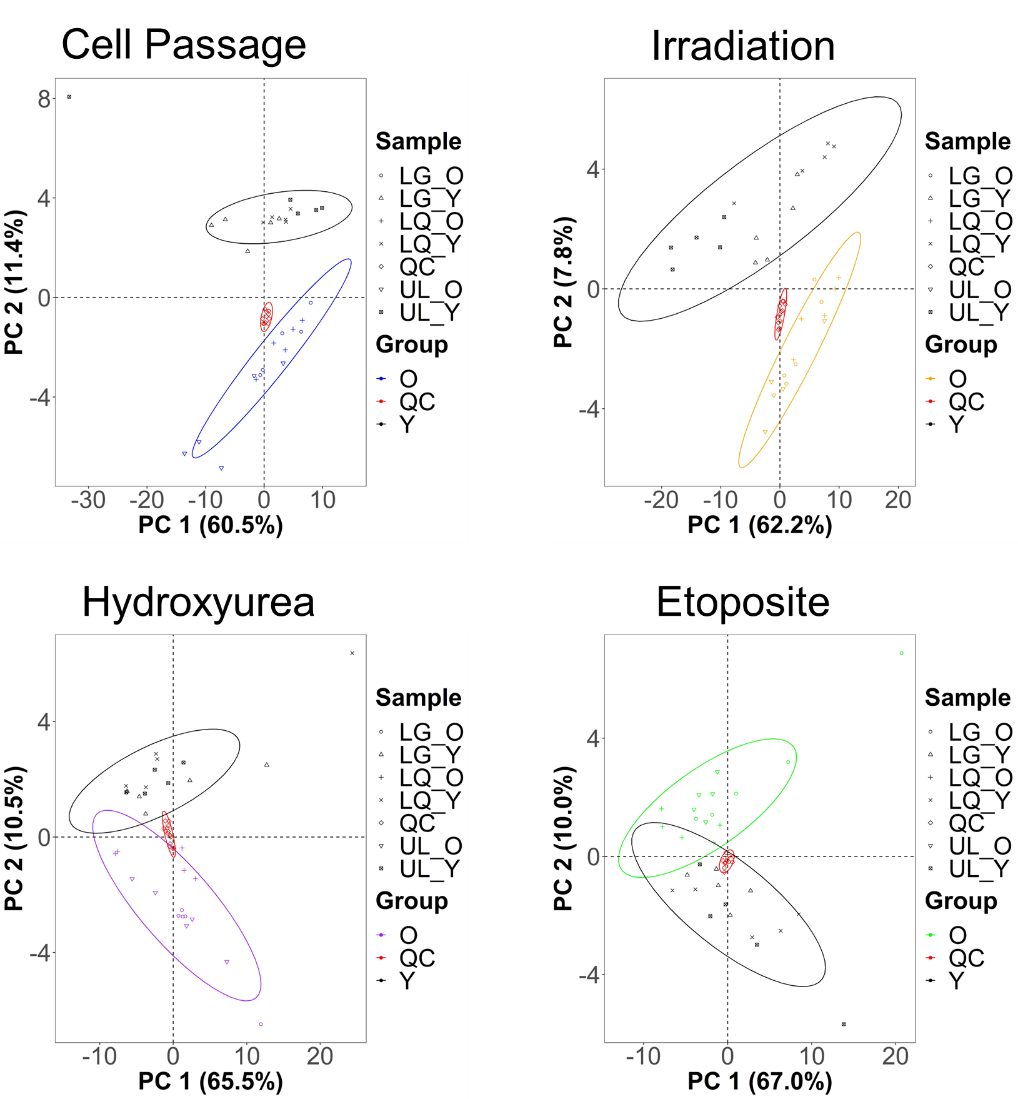


**Supplementary Figure 6|** PCA analysis of global metabolites both labeled and non-labeled with [^13^C_6_]-glucose and [^13^C_5_, ^15^N_2_]-glutamine in different senescent-induced cells. For each treatment group, five replicates were used. Data points in the two-dimensional PCA score plot were central scaled.


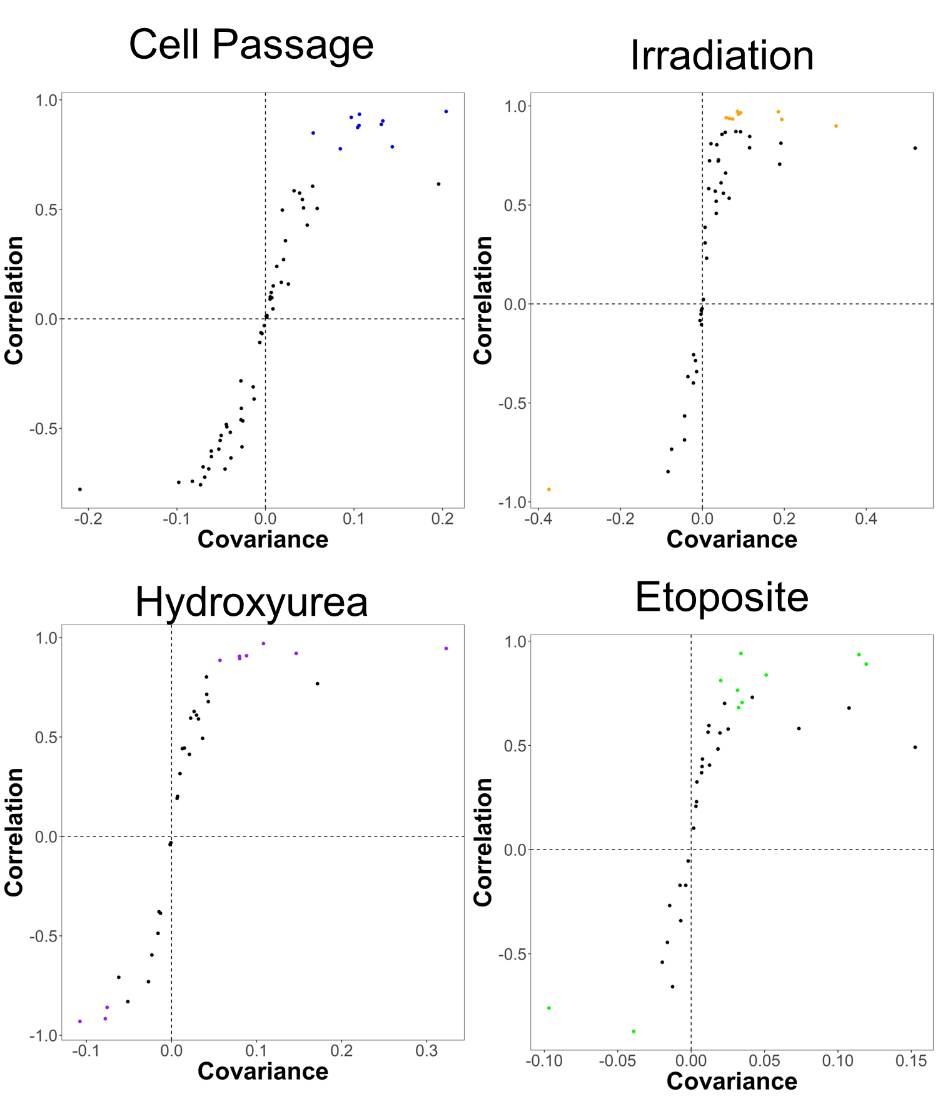


**Supplementary Figure 7**| Partial Least Square Discriminant Analysis (PLS-DA) of metabolomics data from cells at passage 20, cells treated with 12 Gy irradiation, 800 µM hydroxyurea and 10 µM etoposide. Colored data points represent the first 10 discriminating compounds which mainly contribute to the separation between treated and non-treated cells.

**Supplementary Table 10|** List of metabolites identified after PLS-DA analysis for each tested conditions including cells at passage 20, cells treated with 12 Gy irradiation, 800 µM hydroxyurea and 10 µM etoposide.

| Cell Passage |  |  |  |  |  |
| --- | --- | --- | --- | --- | --- |
| **Name** | **Mass** | **ppm** | **Rt** | **Log2 fold change** | **p-value** |
| Proline | 115.0633 | 0.02 | 2.73 | -1.32 | 2.40E-05 |
| Taurine | 125.01466 | -0.01 | 4.47 | -1.26 | 1.44E-04 |
| Creatine | 131.06948 | -0.52 | 3.77 | -0.89 | 1.31E-02 |
| Deoxycarnitine | 145.11028 | -0.26 | 2.5 | -0.54 | 3.47E-03 |
| Glycine | 75.03203 | -0.21 | 4.59 | -0.88 | 9.61E-04 |
| Alanine | 89.04768 | -0.58 | 3.96 | -0.91 | 1.52E-03 |
| 3-Methyl-2-oxovalerate | 130.06299 | 0.18 | 0.99 | -0.89 | 1.36E-02 |
| Hypotaurine | 109.01975 | 0.28 | 4.16 | -1.09 | 8.67E-04 |
| Malate | 134.02152 | 0.27 | 2.86 | -1.41 | 8.82E-04 |
| Asparagine | 132.05349 | -0.18 | 4.75 | -1.8 | 1.50E-06 |
| Irradiation |  |  |  |  |  |
| **Name** | **Mass** | **ppm** | **Rt** | **Log2 fold change** | **p-value** |
| Proline | 115.06336 | 0.27 | 2.76 | -0.67 | 1.11E-04 |
| Carnitine | 161.105516 | -0.19 | 2.97 | -0.43 | 3.46E-05 |
| Glutamate | 147.05316 | -0.01 | 4.76 | -0.54 | 1.56E-04 |
| Phosphocholine | 183.06599 | -0.29 | 4.48 | -0.62 | 9.10E-06 |
| Pantothenate | 219.11064 | -0.17 | 1.05 | -0.43 | 1.97E-04 |
| Bis(2-ethylhexyl)phthalate | 390.27695 | -0.15 | 0.67 | 2.28 | 6.42E-04 |
| Taurine | 125.01467 | 0.08 | 4.48 | -1.44 | 4.77E-07 |
| Deoxycarnitine | 145.11025 | -0.18 | 2.52 | -0.56 | 5.98E-04 |
| O-Phosphoethanolamine | 141.01906 | -0.26 | 5.68 | -1.4 | 4.79E-05 |
| Gluconate | 196.05834 | 0.18 | 4.55 | -2.15 | 1.88E-04 |
| Hydroxyurea |  |  |  |  |  |
| **Name** | **Mass** | **ppm** | **Rt** | **Log2 fold change** | **p-value** |
| Glutamine | 146.06914 | -0.04 | 4.54 | 0.68 | 1.07E-02 |
| Proline | 115.06333 | 0.13 | 2.79 | -0.89 | 2.14E-04 |
| Glutathione | 307.08381 | 0.06 | 4.93 | 0.71 | 2.43E-04 |
| Lactate | 90.03169 | 0.56 | 1.44 | -0.83 | 4.41E-05 |
| Valine | 117.07896 | -0.14 | 2.07 | -1.08 | 3.30E-05 |
| Taurine | 125.01466 | -0.04 | 4.54 | -1.44 | 3.97E-05 |
| Serine | 105.04257 | -0.22 | 4.89 | 1.02 | 1.02E-04 |
| Deoxycarnitine | 145.11025 | -0.19 | 2.54 | -0.68 | 2.27E-04 |
| O-Acetylcarnitine | 203.11565 | -0.51 | 1.79 | -2.79 | 5.62E-03 |
| Mevalolactone | 130.06302 | 0.16 | 1.02 | -0.94 | 1.18E-05 |
| Etoposide |  |  |  |  |  |
| **Name** | **Mass** | **ppm** | **Rt** | **Log2 fold change** | **p-value** |
| Gulose | 180.06339 | 0.23 | 3.46 | -0.47 | 1.46E-02 |
| Carnitine | 161.10519 | -0.19 | 2.99 | -0.59 | 7.20E-04 |
| Glucose | 180.06339 | 0.22 | 3.79 | -0.45 | 1.95E-02 |
| Valine | 117.07898 | -0.05 | 2.84 | -0.34 | 8.41E-03 |
| Norvaline | 117.07898 | -0.13 | 2.04 | -0.30 | 1.96E-02 |
| Taurine | 125.01466 | 0.12 | 4.53 | -1.35 | 1.64E-04 |
| O-Acetylcarnitine | 203.11576 | -0.51 | 1.86 | 1.25 | 2.06E-03 |
| Deoxycarnitine | 145.11028 | -0.26 | 2.54 | -0.43 | 1.75E-07 |
| 3-Methyl-2-oxovalerate | 130.06299 | 0.18 | 1.03 | -1.52 | 7.58E-04 |
| Serine | 105.04259 | 0.11 | 4.88 | 0.53 | 2.65E-04 |

**Supplementary Table 11|** List of metabolites identified in the media of each tested conditions including cells at passage 20, cells treated with 12 Gy irradiation, 800 µM hydroxyurea and 10 µM etoposide

| Cell passage |  |  |  |  |  |
| --- | --- | --- | --- | --- | --- |
| **Name** | **Mass** | **ppm** | **rt** | **Log2 Fold change** | **p-value** |
| METHYL ACETOACETATE | 115.0401 | 0.23 | 1.21 | -1.14 | 4.45E-06 |
| HYPOXANTHINE | 137.0457 | -0.56 | 1.75 | 4.47 | 6.36E-06 |
| MEVALOLACTONE | 129.0558 | 0.23 | 1.01 | -0.75 | 1.19E-05 |
| INDOLEACETALDEHYDE | 160.0756 | -0.52 | 0.99 | 1.3 | 3.27E-05 |
| CYSTINE | 241.031 | -0.6 | 6.81 | 0.31 | 1.66E-03 |
| PYRUVATE | 87.00879 | 0.28 | 1.97 | -0.38 | 7.85E-03 |
| LACTATE | 89.02447 | 0.62 | 1.41 | -0.58 | 1.36E-02 |
| GLYCERATE | 87.0088 | 0.35 | 1.98 | -0.38 | 1.79E-02 |
| PROLINE | 116.0706 | -0.23 | 2.72 | -0.77 | 3.79E-02 |
| Irradiation |  |  |  |  |  |
| **Name** | **Mass** | **ppm** | **rt** | **Log2 Fold change** | **p-value** |
| D-ORNITHINE | 133.0971 | -0.35 | 8.85 | -0.39 | 1.29E-03 |
| HYPOXANTHINE | 137.0457 | -0.77 | 1.79 | 3.01 | 1.43E-03 |
| METHYL ACETOACETATE | 115.0401 | 0.36 | 1.23 | -0.58 | 1.75E-03 |
| DEOXYURIDINE | 263.044 | -0.11 | 1.28 | 0.86 | 1.95E-03 |
| MEVALOLACTONE | 129.0558 | 0.33 | 1.01 | -0.42 | 2.26E-03 |
| DEOXYURIDINE-MONOPHOSPHATE | 307.0348 | 3.49 | 1.43 | -0.56 | 2.98E-03 |
| SERINE | 106.0498 | -0.35 | 4.84 | 0.56 | 6.24E-03 |
| CYSTINE | 241.031 | -0.37 | 6.85 | 0.4 | 1.86E-02 |
| LACTATE | 89.02447 | 0.6 | 1.43 | -0.69 | 3.09E-02 |
| CYSTEATE | 211.0389 | 3.25 | 1.44 | -0.39 | 3.35E-02 |
| DEOXYCYTIDINE | 266.0535 | -0.74 | 2.12 | -1.71 | 3.93E-02 |
| Hydroxyurea |  |  |  |  |  |
| **Name** | **Mass** | **ppm** | **rt** | **Log2 Fold change** | **p-value** |
| DEOXYCYTIDINE | 228.0977 | -0.79 | 1.25 | 2.77 | 2.70E-05 |
| INDOLEACETALDEHYDE | 160.0756 | -0.84 | 0.97 | 1.33 | 2.90E-05 |
| METHYL ACETOACETATE | 115.0401 | 0.23 | 1.18 | -0.87 | 1.00E-03 |
| UROCANATE | 139.0501 | -0.89 | 1.46 | 0.49 | 1.51E-03 |
| 4-HYDROXY-L-PHENYLGLYCINE | 168.0654 | -0.69 | 1.31 | -0.55 | 3.20E-03 |
| MEVALOLACTONE | 129.0558 | 0.25 | 0.99 | -0.76 | 1.15E-02 |
| DOCOSAHEXAENOATE | 327.233 | 0.08 | 0.70 | 1.73 | 1.69E-02 |
| D-ORNITHINE | 133.0971 | -0.56 | 8.78 | -0.62 | 3.07E-02 |
| LACTATE | 89.02447 | 0.58 | 1.40 | -0.51 | 4.72E-02 |
| Etoposide |  |  |  |  |  |
| **Name** | **Mass** | **ppm** | **rt** | **Log2 Fold change** | **p-value** |
| CYTIDINE | 244.0926 | -0.93 | 2.6 | 1.35 | 5.86E-03 |
| PYRUVATE | 87.00881 | 0.44 | 1.9 | 0.4 | 8.44E-03 |
| INDOLEACETALDEHYDE | 160.0756 | -0.76 | 1.0 | 1.47 | 1.10E-02 |
| TAURINE | 126.0219 | -0.18 | 4.5 | 0.25 | 1.23E-02 |
| METHYL ACETOACETATE | 115.0401 | 0.22 | 1.2 | -0.3 | 1.62E-02 |
| PROLINE | 116.0706 | -0.3 | 2.7 | 0.36 | 1.66E-02 |
| SERINE | 106.0498 | -0.41 | 4.8 | 0.22 | 2.87E-02 |
| 4-PYRIDOXATE | 182.0459 | -0.19 | 1.1 | 0.17 | 2.98E-02 |
| TRIGONELLINE | 182.0459 | -0.04 | 1.1 | 0.17 | 2.98E-02 |
| MEVALOLACTONE | 129.0558 | 0.28 | 1.0 | -0.26 | 3.02E-02 |

**Supplementary Table 12|** List of significant proteins identified for cells at passage 20, cells treated with 12 Gy irradiation, 800 µM hydroxyurea and 10 µM etoposide.

| Cell passage |  |  |  |  |  |
| --- | --- | --- | --- | --- | --- |
| Name | kDa | N° AAs | Coverage | Log 2 Ratio | p-value |
| CNN1 | 33.2 | 297 | 39 | 1.65 | 1.00E-15 |
| NIBAN2 | 84.1 | 746 | 39 | -1.34 | 1.00E-15 |
| EIF4A1 | 46.1 | 406 | 41 | -1.33 | 1.00E-15 |
| RACK1 | 35.1 | 317 | 33 | -1.5 | 1.00E-15 |
| RPL6 | 32.7 | 288 | 40 | -0.9 | 1.00E-15 |
| GNG12 | 8 | 72 | 47 | 1.71 | 1.00E-15 |
| RPS28 | 7.8 | 69 | 30 | -1.48 | 1.00E-15 |
| SND1 | 101.9 | 910 | 33 | -1.14 | 1.95E-13 |
| DPP4 | 88.2 | 766 | 33 | 1.06 | 1.09E-11 |
| UBE2L3 | 17.9 | 154 | 46 | -2.37 | 1.22E-11 |
| RPSA | 32.8 | 295 | 37 | -1.37 | 3.44E-11 |
| RAN | 24.4 | 216 | 41 | -1.24 | 4.47E-11 |
| SEPTIN7 | 50.6 | 437 | 35 | -0.82 | 4.58E-11 |
| GDI2 | 50.6 | 445 | 49 | -1.31 | 2.03E-10 |
| HNRNPH1 | 49.2 | 449 | 31 | -0.78 | 4.69E-10 |
| SEPTIN11 | 49.4 | 429 | 33 | -0.57 | 8.63E-10 |
| PCBP1 | 37.5 | 356 | 34 | -1.21 | 2.32E-09 |
| EIF5A | 16.8 | 154 | 41 | -1.56 | 2.91E-09 |
| ARPC5 | 16.3 | 151 | 46 | -0.56 | 4.33E-09 |
| PGRMC2 | 23.8 | 223 | 32 | 1.37 | 8.24E-09 |
| CCT2 | 57.5 | 535 | 34 | -0.9 | 8.43E-09 |
| UGDH | 55 | 494 | 31 | -1.34 | 8.73E-09 |
| CAVIN3 | 27.7 | 261 | 43 | 0.46 | 1.19E-08 |
| CFL1 | 18.5 | 166 | 42 | -1.05 | 1.55E-08 |
| SH3BGRL3 | 10.4 | 93 | 31 | -1.61 | 2.65E-08 |
| EHD2 | 61.1 | 543 | 45 | -0.85 | 3.12E-08 |
| TCP1 | 60.3 | 556 | 31 | -0.76 | 3.15E-08 |
| PSME1 | 28.7 | 249 | 36 | -0.98 | 3.30E-08 |
| HSP90AB1 | 83.2 | 724 | 41 | -0.94 | 4.21E-08 |
| UFM1 | 9.1 | 85 | 51 | -0.99 | 4.75E-08 |
| KRT18 | 48 | 430 | 49 | 1.89 | 6.33E-08 |
| MYOF | 234.6 | 2061 | 43 | 0.6 | 1.08E-07 |
| HSP90AA1 | 84.6 | 732 | 41 | -0.82 | 1.09E-07 |
| TLN1 | 269.6 | 2541 | 45 | -1.09 | 1.15E-07 |
| LDHA | 36.7 | 332 | 45 | -0.89 | 1.16E-07 |
| AKAP12 | 191.4 | 1782 | 31 | 0.85 | 1.33E-07 |
| EEF2 | 95.3 | 858 | 32 | -0.85 | 1.48E-07 |
| CLIC4 | 28.8 | 253 | 38 | -1.02 | 1.58E-07 |
| TUBB3 | 50.4 | 450 | 38 | -1.46 | 2.29E-07 |
| ENO1 | 47.1 | 434 | 53 | -1.19 | 2.34E-07 |
| EHD1 | 60.6 | 534 | 41 | -0.76 | 2.62E-07 |
| TPM1 | 32.7 | 284 | 38 | 0.99 | 3.77E-07 |
| SEPTIN2 | 41.5 | 361 | 49 | -0.68 | 4.02E-07 |
| LAMTOR1 | 17.7 | 161 | 48 | 0.78 | 5.25E-07 |
| FSCN1 | 54.5 | 493 | 30 | -1.35 | 6.29E-07 |
| UBE2N | 17.1 | 152 | 37 | -1.53 | 7.78E-07 |
| WDR1 | 66.2 | 606 | 44 | -1.04 | 9.14E-07 |
| RPS15A | 14.8 | 130 | 36 | -0.5 | 9.25E-07 |
| DBI | 10 | 87 | 51 | -1.9 | 1.00E-06 |
| IQGAP1 | 189.1 | 1657 | 41 | -0.5 | 1.17E-06 |
| ANXA2 | 38.6 | 339 | 55 | -0.64 | 1.49E-06 |
| UBE2V1 | 16.5 | 147 | 34 | -0.88 | 1.50E-06 |
| MSN | 67.8 | 577 | 45 | -0.83 | 1.60E-06 |
| ARF3 | 20.6 | 181 | 54 | -0.71 | 1.83E-06 |
| RPS8 | 24.2 | 208 | 33 | -0.48 | 1.90E-06 |
| YWHAQ | 27.7 | 245 | 40 | -1.19 | 1.92E-06 |
| TUBB | 49.6 | 444 | 44 | -0.97 | 2.04E-06 |
| NME1 | 17.1 | 152 | 53 | -0.7 | 2.41E-06 |
| ALDOA | 39.4 | 364 | 54 | -0.8 | 2.62E-06 |
| PCYOX1 | 56.6 | 505 | 34 | 1.29 | 2.64E-06 |
| PLIN3 | 47 | 434 | 51 | -0.77 | 3.62E-06 |
| BTF3L4 | 17.3 | 158 | 39 | -1.01 | 3.86E-06 |
| HNRNPK | 50.9 | 463 | 40 | -0.75 | 4.48E-06 |
| FERMT2 | 77.8 | 680 | 33 | -0.71 | 5.05E-06 |
| HSPA1B | 70 | 641 | 44 | -0.62 | 5.31E-06 |
| CCT6A | 58 | 531 | 35 | -0.71 | 5.77E-06 |
| CCT4 | 57.9 | 539 | 32 | -0.67 | 6.00E-06 |
| PKM | 57.9 | 531 | 49 | -0.65 | 6.42E-06 |
| RPL10A | 24.8 | 217 | 31 | -0.36 | 6.60E-06 |
| TUBA1B | 50.1 | 451 | 43 | -0.86 | 6.76E-06 |
| EEF1A1 | 50.1 | 462 | 32 | -0.71 | 6.92E-06 |
| CLIC1 | 26.9 | 241 | 32 | -0.93 | 7.41E-06 |
| VAMP2 | 12.7 | 116 | 34 | 0.67 | 7.79E-06 |
| HSPA2 | 70 | 639 | 37 | -1.08 | 9.29E-06 |
| EEF1B2 | 24.7 | 225 | 39 | -0.59 | 1.07E-05 |
| ANPEP | 109.5 | 967 | 41 | 0.67 | 1.27E-05 |
| RPL23A | 17.7 | 156 | 32 | -0.33 | 1.34E-05 |
| CCT3 | 60.5 | 545 | 31 | -0.78 | 1.62E-05 |
| CAVIN1 | 43.5 | 390 | 37 | 0.54 | 1.64E-05 |
| RAB23 | 26.6 | 237 | 31 | 0.54 | 1.66E-05 |
| ARL8B | 21.5 | 186 | 35 | 0.61 | 1.71E-05 |
| VCL | 123.7 | 1134 | 54 | -0.91 | 1.74E-05 |
| PARK7 | 19.9 | 189 | 44 | -0.72 | 1.81E-05 |
| PFN1 | 15 | 140 | 63 | -0.93 | 1.90E-05 |
| TPT1 | 19.6 | 172 | 35 | -1.07 | 1.94E-05 |
| ALYREF | 26.9 | 257 | 33 | -0.4 | 2.37E-05 |
| H2AZ1 | 13.5 | 128 | 54 | -1.12 | 2.38E-05 |
| PEBP1 | 21 | 187 | 59 | -0.86 | 3.25E-05 |
| TUBB4B | 49.8 | 445 | 44 | -1.04 | 3.59E-05 |
| DPYSL2 | 62.3 | 572 | 55 | -0.98 | 3.98E-05 |
| PGAM1 | 28.8 | 254 | 55 | -0.73 | 4.04E-05 |
| RAB6A | 23.6 | 208 | 32 | 0.72 | 4.30E-05 |
| HSPA8 | 70.9 | 646 | 46 | -0.44 | 5.35E-05 |
| VIM | 53.6 | 466 | 80 | 0.66 | 6.12E-05 |
| GPI | 63.1 | 558 | 35 | -0.89 | 6.47E-05 |
| MTPN | 12.9 | 118 | 35 | -0.73 | 7.25E-05 |
| TAGLN2 | 22.4 | 199 | 56 | -0.97 | 8.13E-05 |
| PGK1 | 44.6 | 417 | 39 | -0.71 | 8.25E-05 |
| ELOC | 12.5 | 112 | 37 | -0.54 | 0.0001 |
| RPL7 | 29.2 | 248 | 40 | -0.37 | 0.000105 |
| GSN | 85.6 | 782 | 30 | -0.62 | 0.000113 |
| PSMA2 | 25.9 | 234 | 35 | -0.97 | 0.000114 |
| YWHAB | 28.1 | 246 | 48 | -0.68 | 0.000118 |
| ATP5PD | 18.5 | 161 | 41 | 0.78 | 0.000145 |
| VCP | 89.3 | 806 | 39 | -0.41 | 0.000158 |
| LASP1 | 29.7 | 261 | 37 | -0.88 | 0.000165 |
| ANXA6 | 75.8 | 673 | 55 | -0.87 | 0.000175 |
| RPS2 | 31.3 | 293 | 39 | -0.49 | 0.000189 |
| EEF1G | 50.1 | 437 | 35 | -0.53 | 0.00022 |
| RPL27 | 15.8 | 136 | 30 | -0.38 | 0.000282 |
| PRDX3 | 27.7 | 256 | 31 | 0.84 | 0.000296 |
| HBA1 | 15.2 | 142 | 43 | 1.27 | 0.000305 |
| S100A11 | 11.7 | 105 | 39 | -0.73 | 0.000308 |
| XRCC5 | 82.7 | 732 | 40 | -0.73 | 0.000313 |
| TUBB6 | 49.8 | 446 | 49 | -0.73 | 0.000327 |
| PRDX6 | 25 | 224 | 46 | -1.03 | 0.000344 |
| YWHAZ | 27.7 | 245 | 47 | -0.71 | 0.000364 |
| CCT5 | 59.6 | 541 | 37 | -0.57 | 0.00041 |
| CFL2 | 18.7 | 166 | 33 | -0.92 | 0.000441 |
| XRCC6 | 69.8 | 609 | 35 | -0.52 | 0.000467 |
| CBR1 | 30.4 | 277 | 32 | -0.68 | 0.000493 |
| EEF1D | 31.1 | 281 | 44 | -0.45 | 0.000493 |
| YWHAE | 29.2 | 255 | 60 | -0.62 | 0.000521 |
| MVP | 99.3 | 893 | 49 | -0.42 | 0.000612 |
| RPS14 | 16.3 | 151 | 36 | -0.36 | 0.000618 |
| RAB7A | 23.5 | 207 | 64 | 0.4 | 0.000665 |
| PRDX1 | 22.1 | 199 | 46 | -0.55 | 0.000688 |
| ANXA4 | 35.9 | 319 | 41 | -0.43 | 0.000692 |
| RAB5B | 23.7 | 215 | 36 | 1.2 | 0.00073 |
| TPI1 | 26.7 | 249 | 51 | -0.67 | 0.000826 |
| LDHB | 36.6 | 334 | 31 | -0.55 | 0.00098 |
| ACTN4 | 104.8 | 911 | 56 | -0.6 | 0.001082 |
| PSMB4 | 29.2 | 264 | 38 | -0.53 | 0.00118 |
| RPS18 | 17.7 | 152 | 44 | -0.36 | 0.001233 |
| UBA1 | 117.8 | 1058 | 32 | -0.62 | 0.001268 |
| LAMTOR3 | 13.6 | 124 | 49 | 0.75 | 0.001336 |
| PLEC | 531.5 | 4684 | 48 | 0.34 | 0.001359 |
| RRAS | 23.5 | 218 | 33 | 0.55 | 0.001589 |
| CAV1 | 20.5 | 178 | 51 | 0.66 | 0.001763 |
| RCN2 | 36.9 | 317 | 43 | 1.01 | 0.001781 |
| ATP5ME | 7.9 | 69 | 32 | 0.65 | 0.001795 |
| LGALS1 | 14.7 | 135 | 41 | -0.81 | 0.002137 |
| TPM2 | 32.8 | 284 | 38 | 0.57 | 0.00235 |
| MYH10 | 228.9 | 1976 | 36 | 0.41 | 0.002915 |
| DPYSL3 | 61.9 | 570 | 49 | -1.36 | 0.002975 |
| H4C1 | 11.4 | 103 | 51 | -0.84 | 0.003243 |
| RPL9 | 21.9 | 192 | 48 | -0.5 | 0.004288 |
| SRSF1 | 27.7 | 248 | 35 | -0.45 | 0.004557 |
| RPS4X | 29.6 | 263 | 41 | -0.25 | 0.005239 |
| PSMA1 | 29.5 | 263 | 37 | -0.45 | 0.005806 |
| RPL26 | 17.2 | 145 | 33 | -0.22 | 0.005902 |
| RPS13 | 17.2 | 151 | 34 | -0.41 | 0.006034 |
| CYB5R3 | 34.2 | 301 | 53 | 0.56 | 0.006099 |
| ANXA5 | 35.9 | 320 | 66 | -0.61 | 0.006726 |
| PSMA5 | 26.4 | 241 | 35 | -0.38 | 0.007097 |
| PSMC6 | 44.1 | 389 | 31 | -0.74 | 0.007723 |
| APMAP | 46.5 | 416 | 37 | 0.77 | 0.007877 |
| MYO1C | 121.6 | 1063 | 33 | 0.27 | 0.008143 |
| FLOT2 | 47 | 428 | 32 | 0.64 | 0.008291 |
| PDLIM1 | 36 | 329 | 38 | -0.46 | 0.008767 |
| VAMP3 | 11.3 | 100 | 40 | 1.11 | 0.00907 |
| GSTP1 | 23.3 | 210 | 53 | -0.56 | 0.009158 |
| ATP5F1D | 17.5 | 168 | 38 | 0.51 | 0.009567 |
| YWHAH | 28.2 | 246 | 36 | -0.88 | 0.009576 |
| PSMA6 | 27.4 | 246 | 35 | -0.45 | 0.00984 |
| RPS3 | 26.7 | 243 | 51 | -0.3 | 0.010531 |
| S100A4 | 11.7 | 101 | 36 | -0.73 | 0.01088 |
| PSMA7 | 27.9 | 248 | 33 | -0.44 | 0.011681 |
| CALU | 37.1 | 315 | 63 | 0.6 | 0.012217 |
| CAP1 | 51.9 | 475 | 35 | -0.53 | 0.012367 |
| RPS3A | 29.9 | 264 | 33 | -0.33 | 0.014841 |
| ATP5F1A | 59.7 | 553 | 44 | 0.51 | 0.016235 |
| VDAC1 | 30.8 | 283 | 57 | 0.56 | 0.017961 |
| TKT | 67.8 | 623 | 40 | -0.54 | 0.018461 |
| MAP4 | 120.9 | 1152 | 30 | -0.44 | 0.01945 |
| RAP1B | 20.8 | 184 | 34 | 0.27 | 0.020983 |
| PSMB1 | 26.5 | 241 | 39 | -0.4 | 0.021074 |
| HSD17B12 | 34.3 | 312 | 36 | 0.71 | 0.02114 |
| RPS16 | 16.4 | 146 | 47 | -0.29 | 0.023844 |
| H2AX | 15.1 | 143 | 33 | -0.64 | 0.025394 |
| HSPA9 | 73.6 | 679 | 47 | 0.58 | 0.025904 |
| ETFA | 35.1 | 333 | 39 | 0.8 | 0.026311 |
| ARF4 | 20.5 | 180 | 42 | -0.37 | 0.02655 |
| NT5E | 63.3 | 574 | 38 | 0.7 | 0.029969 |
| PRDX2 | 21.9 | 198 | 32 | -0.52 | 0.031435 |
| RPS17 | 15.5 | 135 | 47 | -0.4 | 0.032884 |
| ACTN1 | 103 | 892 | 51 | -0.3 | 0.033029 |
| VAT1 | 41.9 | 393 | 34 | 0.21 | 0.033363 |
| HADHA | 82.9 | 763 | 43 | 0.57 | 0.033796 |
| MYH9 | 226.4 | 1960 | 50 | 0.26 | 0.037891 |
| ATP5F1B | 56.5 | 529 | 67 | 0.49 | 0.03807 |
| MACROH2A1 | 39.2 | 369 | 35 | 0.3 | 0.038205 |
| GAPDH | 36 | 335 | 61 | -0.85 | 0.038608 |
| RPLP2 | 11.7 | 115 | 77 | -0.34 | 0.043683 |
| IKBIP | 39.3 | 350 | 37 | 0.77 | 0.04447 |
| Irradiation |  |  |  |  |  |
| Name | kDa | N° AAs | Coverage | Log 2 Ratio | p-value |
| ARPC2 | 34.3 | 300 | 57 | -0.4 | 5.09E-09 |
| RAN | 24.4 | 216 | 41 | -0.98 | 2.21E-08 |
| ARPC5 | 16.3 | 151 | 46 | -0.45 | 1.84E-07 |
| MYOF | 234.6 | 2061 | 43 | 0.53 | 2.90E-07 |
| GDI2 | 50.6 | 445 | 49 | -0.85 | 7.55E-07 |
| CYB5B | 16.7 | 150 | 43 | 1.35 | 9.99E-07 |
| DBI | 10 | 87 | 51 | -1.56 | 1.04E-06 |
| ANXA2 | 38.6 | 339 | 55 | -0.67 | 1.54E-06 |
| DPYSL3 | 61.9 | 570 | 49 | -1.43 | 1.66E-05 |
| H2AZ1 | 13.5 | 128 | 54 | -0.91 | 1.83E-05 |
| H2AX | 15.1 | 143 | 33 | -0.89 | 2.47E-05 |
| CYB5R3 | 34.2 | 301 | 53 | 0.65 | 2.77E-05 |
| UBE2V1 | 16.5 | 147 | 34 | -0.87 | 3.39E-05 |
| UGDH | 55 | 494 | 31 | -0.83 | 3.45E-05 |
| VIM | 53.6 | 466 | 80 | 0.53 | 4.95E-05 |
| H2BC12 | 13.9 | 126 | 47 | -0.93 | 5.39E-05 |
| FSCN1 | 54.5 | 493 | 30 | -0.97 | 7.68E-05 |
| ANXA4 | 35.9 | 319 | 41 | -0.54 | 0.000102 |
| SEPTIN11 | 49.4 | 429 | 33 | -0.38 | 0.000131 |
| RCN2 | 36.9 | 317 | 43 | 0.84 | 0.000137 |
| CBR1 | 30.4 | 277 | 32 | -0.96 | 0.000141 |
| LSM2 | 10.8 | 95 | 32 | -0.84 | 0.000221 |
| PCBP1 | 37.5 | 356 | 34 | -0.67 | 0.000238 |
| PGRMC2 | 23.8 | 223 | 32 | 0.73 | 0.000244 |
| ENO1 | 47.1 | 434 | 53 | -0.86 | 0.000245 |
| PARK7 | 19.9 | 189 | 44 | -0.9 | 0.000255 |
| KRT18 | 48 | 430 | 49 | 1.03 | 0.000323 |
| H4C1 | 11.4 | 103 | 51 | -0.78 | 0.000393 |
| SH3BGRL3 | 10.4 | 93 | 31 | -0.77 | 0.000414 |
| MTPN | 12.9 | 118 | 35 | -0.81 | 0.000446 |
| EEF2 | 95.3 | 858 | 32 | -0.65 | 0.000506 |
| VCL | 123.7 | 1134 | 54 | -0.8 | 0.000555 |
| EIF5A | 16.8 | 154 | 41 | -0.81 | 0.000559 |
| CFL1 | 18.5 | 166 | 42 | -0.7 | 0.00061 |
| MYH10 | 228.9 | 1976 | 36 | -0.63 | 0.000657 |
| TPI1 | 26.7 | 249 | 51 | -0.83 | 0.000666 |
| S100A4 | 11.7 | 101 | 36 | -0.75 | 0.000672 |
| HSP90AA1 | 84.6 | 732 | 41 | -0.55 | 0.0007 |
| LMNB1 | 66.4 | 586 | 34 | -0.85 | 0.000744 |
| EIF4A1 | 46.1 | 406 | 41 | -0.52 | 0.000773 |
| ANXA6 | 75.8 | 673 | 55 | -0.67 | 0.000874 |
| MSN | 67.8 | 577 | 45 | -0.54 | 0.00089 |
| SQOR | 49.9 | 450 | 45 | 0.58 | 0.000901 |
| HSP90AB1 | 83.2 | 724 | 41 | -0.56 | 0.000905 |
| NES | 177.3 | 1621 | 34 | 0.58 | 0.001035 |
| TUBB | 49.6 | 444 | 44 | -0.7 | 0.001056 |
| TUBA1B | 50.1 | 451 | 43 | -0.6 | 0.001081 |
| UBE2L3 | 17.9 | 154 | 46 | -0.8 | 0.001112 |
| IFITM3 | 14.6 | 133 | 31 | -0.52 | 0.001244 |
| CNN1 | 33.2 | 297 | 39 | 0.41 | 0.001311 |
| HADHA | 82.9 | 763 | 43 | 0.6 | 0.001325 |
| DPYSL2 | 62.3 | 572 | 55 | -0.61 | 0.001344 |
| HSD17B12 | 34.3 | 312 | 36 | 0.67 | 0.001346 |
| H2AC4 | 14.1 | 130 | 33 | -0.81 | 0.00138 |
| CANX | 67.5 | 592 | 45 | 0.64 | 0.001435 |
| LDHA | 36.7 | 332 | 45 | -0.62 | 0.001482 |
| BTF3L4 | 17.3 | 158 | 39 | -0.74 | 0.001489 |
| NT5E | 63.3 | 574 | 38 | 0.64 | 0.001514 |
| RAB5C | 23.5 | 216 | 48 | 0.49 | 0.001615 |
| LASP1 | 29.7 | 261 | 37 | -0.74 | 0.001783 |
| EEF1A1 | 50.1 | 462 | 32 | -0.44 | 0.002195 |
| TLN1 | 269.6 | 2541 | 45 | -0.5 | 0.002263 |
| YWHAB | 28.1 | 246 | 48 | -0.74 | 0.002339 |
| CNPY2 | 20.6 | 182 | 37 | 0.82 | 0.002349 |
| GSN | 85.6 | 782 | 30 | -0.59 | 0.002431 |
| ASPH | 85.8 | 758 | 32 | 0.48 | 0.002748 |
| PFN1 | 15 | 140 | 63 | -0.76 | 0.002773 |
| TAGLN | 22.6 | 201 | 58 | -0.72 | 0.002781 |
| YWHAQ | 27.7 | 245 | 40 | -0.67 | 0.002966 |
| PSME1 | 28.7 | 249 | 36 | -0.5 | 0.003115 |
| CAVIN1 | 43.5 | 390 | 37 | 0.36 | 0.003219 |
| YWHAZ | 27.7 | 245 | 47 | -0.73 | 0.003348 |
| PEBP1 | 21 | 187 | 59 | -0.62 | 0.004015 |
| FERMT2 | 77.8 | 680 | 33 | -0.42 | 0.004613 |
| CAP1 | 51.9 | 475 | 35 | -0.66 | 0.004915 |
| CLIC1 | 26.9 | 241 | 32 | -0.79 | 0.00492 |
| HSPB1 | 22.8 | 205 | 77 | -0.67 | 0.00495 |
| IER3IP1 | 9 | 82 | 34 | 0.38 | 0.005834 |
| SSR4 | 19 | 173 | 31 | 0.66 | 0.005877 |
| ETFA | 35.1 | 333 | 39 | 0.67 | 0.007119 |
| LAMTOR3 | 13.6 | 124 | 49 | 0.73 | 0.007227 |
| ATP1A1 | 112.8 | 1023 | 31 | 0.32 | 0.007496 |
| LAMTOR1 | 17.7 | 161 | 48 | 0.39 | 0.007912 |
| LDHB | 36.6 | 334 | 31 | -0.56 | 0.00792 |
| NME1 | 17.1 | 152 | 53 | -0.5 | 0.008071 |
| ATP5F1A | 59.7 | 553 | 44 | 0.39 | 0.008503 |
| PCYOX1 | 56.6 | 505 | 34 | 0.85 | 0.008892 |
| HMGA2 | 11.8 | 109 | 31 | 0.48 | 0.00897 |
| GSTK1 | 25.5 | 226 | 33 | 0.75 | 0.009146 |
| ERLIN2 | 37.8 | 339 | 41 | 0.53 | 0.009501 |
| SEPTIN7 | 50.6 | 437 | 35 | -0.33 | 0.010308 |
| PGAM1 | 28.8 | 254 | 55 | -0.43 | 0.01037 |
| PKM | 57.9 | 531 | 49 | -0.48 | 0.011738 |
| H2BC11 | 13.9 | 126 | 47 | -0.89 | 0.012144 |
| ATP5F1B | 56.5 | 529 | 67 | 0.51 | 0.012507 |
| AK3 | 25.6 | 227 | 43 | 0.82 | 0.012592 |
| OAT | 48.5 | 439 | 38 | 0.82 | 0.013177 |
| RPS19 | 16.1 | 145 | 53 | -0.35 | 0.013366 |
| RPS3 | 26.7 | 243 | 51 | -0.34 | 0.014413 |
| PGK1 | 44.6 | 417 | 39 | -0.55 | 0.014578 |
| HSPA2 | 70 | 639 | 37 | -0.61 | 0.015389 |
| RRAS | 23.5 | 218 | 33 | 0.34 | 0.015777 |
| CKAP4 | 66 | 602 | 56 | 0.55 | 0.015911 |
| S100A11 | 11.7 | 105 | 39 | -0.46 | 0.016676 |
| APMAP | 46.5 | 416 | 37 | 0.46 | 0.017063 |
| SEC22B | 24.7 | 215 | 50 | 0.47 | 0.017826 |
| RPL23A | 17.7 | 156 | 32 | -0.32 | 0.018822 |
| VDAC3 | 30.6 | 283 | 32 | 0.28 | 0.019538 |
| MYH9 | 226.4 | 1960 | 50 | -0.31 | 0.019718 |
| TCP1 | 60.3 | 556 | 31 | -0.54 | 0.020799 |
| TUBB4B | 49.8 | 445 | 44 | -0.6 | 0.023127 |
| ELOC | 12.5 | 112 | 37 | -0.48 | 0.023681 |
| HADHB | 51.3 | 474 | 32 | 0.42 | 0.023703 |
| WDR1 | 66.2 | 606 | 44 | -0.45 | 0.024478 |
| GSTP1 | 23.3 | 210 | 53 | -0.5 | 0.024915 |
| ATP5PF | 12.6 | 108 | 31 | 0.38 | 0.025895 |
| PLIN3 | 47 | 434 | 51 | -0.44 | 0.027322 |
| DPP4 | 88.2 | 766 | 33 | 0.38 | 0.028139 |
| RPS15A | 14.8 | 130 | 36 | -0.22 | 0.028737 |
| YWHAE | 29.2 | 255 | 60 | -0.53 | 0.032767 |
| CCT2 | 57.5 | 535 | 34 | -0.51 | 0.032787 |
| ANXA5 | 35.9 | 320 | 66 | -0.59 | 0.03298 |
| YWHAH | 28.2 | 246 | 36 | -0.86 | 0.033483 |
| ATP5PD | 18.5 | 161 | 41 | 0.38 | 0.038671 |
| CAVIN3 | 27.7 | 261 | 43 | 0.15 | 0.039158 |
| UFM1 | 9.1 | 85 | 51 | -0.43 | 0.040509 |
| RPL26 | 17.2 | 145 | 33 | -0.28 | 0.041329 |
| CLIC4 | 28.8 | 253 | 38 | -0.55 | 0.042146 |
| TUBB6 | 49.8 | 446 | 49 | -0.4 | 0.042778 |
| NIBAN2 | 84.1 | 746 | 39 | -0.43 | 0.04349 |
| MARCKS | 31.5 | 332 | 61 | -0.48 | 0.044455 |
| Hydroxyurea |  |  |  |  |  |
| Name | kDa | N° AAs | Coverage | Log 2 Ratio | p-value |
| ARPC2 | 34.3 | 300 | 57 | -0.5 | 1.00E-15 |
| VIM | 53.6 | 466 | 80 | 0.97 | 1.00E-15 |
| MYH10 | 228.9 | 1976 | 36 | -1.58 | 1.00E-15 |
| NT5E | 63.3 | 574 | 38 | 1.79 | 1.00E-15 |
| HSPA2 | 70 | 639 | 37 | -1.71 | 1.53E-12 |
| NES | 177.3 | 1621 | 34 | 1.15 | 1.13E-11 |
| TLN1 | 269.6 | 2541 | 45 | -1.25 | 2.14E-11 |
| ARPC5 | 16.3 | 151 | 46 | -0.61 | 2.20E-11 |
| ATP5PF | 12.6 | 108 | 31 | 1.03 | 3.31E-11 |
| SEPTIN7 | 50.6 | 437 | 35 | -0.79 | 8.99E-11 |
| VDAC3 | 30.6 | 283 | 32 | 0.59 | 1.68E-10 |
| RPS2 | 31.3 | 293 | 39 | -0.87 | 2.20E-10 |
| UGDH | 55 | 494 | 31 | -1.53 | 2.67E-10 |
| ATP1A1 | 112.8 | 1023 | 31 | 0.78 | 3.14E-10 |
| SEPTIN11 | 49.4 | 429 | 33 | -0.51 | 4.50E-10 |
| MVP | 99.3 | 893 | 49 | -0.97 | 6.71E-10 |
| YWHAH | 28.2 | 246 | 36 | -2.35 | 9.07E-10 |
| PGRMC2 | 23.8 | 223 | 32 | 1.15 | 1.28E-09 |
| ALDH1A1 | 54.8 | 501 | 40 | -2.12 | 1.32E-09 |
| CANX | 67.5 | 592 | 45 | 1.04 | 1.40E-09 |
| APMAP | 46.5 | 416 | 37 | 1.17 | 1.47E-09 |
| VDAC1 | 30.8 | 283 | 57 | 1.01 | 1.73E-09 |
| ANXA6 | 75.8 | 673 | 55 | -1.61 | 2.58E-09 |
| ANXA2 | 38.6 | 339 | 55 | -0.83 | 3.70E-09 |
| MYOF | 234.6 | 2061 | 43 | 0.56 | 4.26E-09 |
| HSPA9 | 73.6 | 679 | 47 | 0.87 | 1.04E-08 |
| ATP5F1A | 59.7 | 553 | 44 | 0.81 | 1.10E-08 |
| ETFA | 35.1 | 333 | 39 | 1.47 | 1.38E-08 |
| TAGLN2 | 22.4 | 199 | 56 | -1.39 | 1.48E-08 |
| NPM1 | 32.6 | 294 | 31 | 0.74 | 1.75E-08 |
| IER3IP1 | 9 | 82 | 34 | 0.75 | 2.14E-08 |
| RPS28 | 7.8 | 69 | 30 | -0.68 | 2.68E-08 |
| RACK1 | 35.1 | 317 | 33 | -0.97 | 2.76E-08 |
| DPYSL2 | 62.3 | 572 | 55 | -1.37 | 3.38E-08 |
| CYB5B | 16.7 | 150 | 43 | 1.86 | 4.82E-08 |
| P4HA1 | 61 | 534 | 46 | 1.12 | 4.97E-08 |
| TAGLN | 22.6 | 201 | 58 | -1.54 | 5.09E-08 |
| HADHB | 51.3 | 474 | 32 | 0.87 | 5.40E-08 |
| GDI2 | 50.6 | 445 | 49 | -1.18 | 5.52E-08 |
| ETFB | 27.8 | 255 | 36 | 0.97 | 5.95E-08 |
| YWHAZ | 27.7 | 245 | 47 | -1.39 | 7.36E-08 |
| HADHA | 82.9 | 763 | 43 | 0.86 | 8.16E-08 |
| CKAP4 | 66 | 602 | 56 | 1.1 | 8.34E-08 |
| IKBIP | 39.3 | 350 | 37 | 1.2 | 8.56E-08 |
| CFL1 | 18.5 | 166 | 42 | -1.12 | 1.02E-07 |
| HSPD1 | 61 | 573 | 58 | 0.78 | 1.09E-07 |
| RAN | 24.4 | 216 | 41 | -0.94 | 1.16E-07 |
| ATP5PD | 18.5 | 161 | 41 | 0.85 | 1.31E-07 |
| EIF4A1 | 46.1 | 406 | 41 | -1.1 | 1.36E-07 |
| BTF3L4 | 17.3 | 158 | 39 | -1.19 | 1.88E-07 |
| DPYSL3 | 61.9 | 570 | 49 | -1.97 | 2.08E-07 |
| UBE2L3 | 17.9 | 154 | 46 | -1.31 | 2.11E-07 |
| RAB23 | 26.6 | 237 | 31 | 0.51 | 2.24E-07 |
| ATP5ME | 7.9 | 69 | 32 | 0.84 | 2.26E-07 |
| TKT | 67.8 | 623 | 40 | -1.19 | 2.74E-07 |
| VCL | 123.7 | 1134 | 54 | -1.24 | 2.75E-07 |
| CNPY2 | 20.6 | 182 | 37 | 1.15 | 3.28E-07 |
| PRDX6 | 25 | 224 | 46 | -1.45 | 3.32E-07 |
| TUBB | 49.6 | 444 | 44 | -1.19 | 3.45E-07 |
| ATP5F1B | 56.5 | 529 | 67 | 0.84 | 3.60E-07 |
| PHB1 | 29.8 | 272 | 49 | 0.6 | 3.79E-07 |
| HSD17B12 | 34.3 | 312 | 36 | 1 | 3.88E-07 |
| CLIC4 | 28.8 | 253 | 38 | -1.32 | 4.43E-07 |
| SSR4 | 19 | 173 | 31 | 0.96 | 5.47E-07 |
| RCN1 | 38.9 | 331 | 68 | 1.21 | 7.80E-07 |
| MSN | 67.8 | 577 | 45 | -1 | 8.51E-07 |
| ASPH | 85.8 | 758 | 32 | 0.7 | 1.07E-06 |
| LASP1 | 29.7 | 261 | 37 | -1.13 | 1.09E-06 |
| TMEM43 | 44.8 | 400 | 35 | 0.97 | 1.14E-06 |
| TUBA1B | 50.1 | 451 | 43 | -1.09 | 1.52E-06 |
| ACAT1 | 45.2 | 427 | 35 | 0.49 | 1.57E-06 |
| CYB5R3 | 34.2 | 301 | 53 | 0.81 | 1.69E-06 |
| SOD2 | 24.7 | 222 | 63 | 1.74 | 1.90E-06 |
| RRBP1 | 152.4 | 1410 | 50 | 0.64 | 2.08E-06 |
| ERLIN2 | 37.8 | 339 | 41 | 0.84 | 2.31E-06 |
| TUFM | 49.8 | 455 | 32 | 0.81 | 2.55E-06 |
| CAP1 | 51.9 | 475 | 35 | -0.95 | 2.80E-06 |
| MLEC | 32.2 | 292 | 37 | 0.72 | 2.80E-06 |
| UFM1 | 9.1 | 85 | 51 | -1.03 | 2.88E-06 |
| LDHB | 36.6 | 334 | 31 | -1.09 | 3.95E-06 |
| GSTK1 | 25.5 | 226 | 33 | 0.95 | 4.26E-06 |
| SEC22B | 24.7 | 215 | 50 | 0.73 | 4.51E-06 |
| GSN | 85.6 | 782 | 30 | -1.06 | 5.40E-06 |
| H2AC4 | 14.1 | 130 | 33 | -1.33 | 5.49E-06 |
| PFN1 | 15 | 140 | 63 | -1.24 | 6.31E-06 |
| SQOR | 49.9 | 450 | 45 | 0.77 | 6.99E-06 |
| HSPB1 | 22.8 | 205 | 77 | -0.96 | 7.09E-06 |
| PPIB | 23.7 | 216 | 44 | 0.69 | 7.82E-06 |
| MME | 85.5 | 750 | 35 | -0.84 | 8.70E-06 |
| RCN3 | 37.5 | 328 | 68 | 0.93 | 9.27E-06 |
| MDH2 | 35.5 | 338 | 40 | 0.85 | 9.27E-06 |
| RPSA | 32.8 | 295 | 37 | -0.91 | 1.01E-05 |
| HSD17B10 | 26.9 | 261 | 49 | 0.63 | 1.02E-05 |
| GSTP1 | 23.3 | 210 | 53 | -1.26 | 1.06E-05 |
| PRDX3 | 27.7 | 256 | 31 | 0.89 | 1.10E-05 |
| ERP29 | 29 | 261 | 44 | 0.63 | 1.20E-05 |
| FLNA | 280.6 | 2647 | 52 | -0.75 | 1.34E-05 |
| PDLIM1 | 36 | 329 | 38 | -0.81 | 1.40E-05 |
| RPS18 | 17.7 | 152 | 44 | -0.69 | 1.57E-05 |
| H4C1 | 11.4 | 103 | 51 | -0.85 | 1.60E-05 |
| RAB6A | 23.6 | 208 | 32 | 0.42 | 1.63E-05 |
| AHNAK | 628.7 | 5890 | 62 | -0.68 | 1.63E-05 |
| CBR1 | 30.4 | 277 | 32 | -1.16 | 1.85E-05 |
| SEPTIN2 | 41.5 | 361 | 49 | -0.66 | 2.29E-05 |
| P4HB | 57.1 | 508 | 67 | 0.92 | 2.33E-05 |
| UBE2V1 | 16.5 | 147 | 34 | -1.08 | 2.51E-05 |
| PHB2 | 33.3 | 299 | 38 | 0.41 | 2.64E-05 |
| PEBP1 | 21 | 187 | 59 | -1.12 | 2.64E-05 |
| FSCN1 | 54.5 | 493 | 30 | -1.37 | 2.69E-05 |
| HSPE1 | 10.9 | 102 | 40 | 0.66 | 2.93E-05 |
| OAT | 48.5 | 439 | 38 | 0.96 | 3.31E-05 |
| ATP5F1D | 17.5 | 168 | 38 | 0.61 | 3.32E-05 |
| LDHA | 36.7 | 332 | 45 | -0.96 | 3.46E-05 |
| WDR1 | 66.2 | 606 | 44 | -1.03 | 3.48E-05 |
| AK2 | 26.5 | 239 | 30 | 0.84 | 3.51E-05 |
| KRT18 | 48 | 430 | 49 | 1.34 | 4.08E-05 |
| FERMT2 | 77.8 | 680 | 33 | -0.72 | 4.31E-05 |
| PCBP1 | 37.5 | 356 | 34 | -0.82 | 4.49E-05 |
| RAB10 | 22.5 | 200 | 35 | 0.64 | 4.51E-05 |
| RPS17 | 15.5 | 135 | 47 | -0.64 | 4.51E-05 |
| CAVIN1 | 43.5 | 390 | 37 | 0.36 | 5.27E-05 |
| ENO1 | 47.1 | 434 | 53 | -1.1 | 5.31E-05 |
| COX4I1 | 19.6 | 169 | 38 | 0.37 | 6.62E-05 |
| MYH9 | 226.4 | 1960 | 50 | -0.46 | 6.88E-05 |
| RPN1 | 68.5 | 607 | 52 | 0.47 | 7.20E-05 |
| H2BC11 | 13.9 | 126 | 47 | -1.73 | 7.30E-05 |
| IMMT | 83.6 | 758 | 32 | 0.59 | 7.40E-05 |
| HMGA2 | 11.8 | 109 | 31 | 0.81 | 7.58E-05 |
| TUBB6 | 49.8 | 446 | 49 | -1.06 | 8.13E-05 |
| YWHAB | 28.1 | 246 | 48 | -0.97 | 8.28E-05 |
| YWHAE | 29.2 | 255 | 60 | -0.99 | 9.94E-05 |
| ACTN4 | 104.8 | 911 | 56 | -0.71 | 0.000108 |
| HSP90AB1 | 83.2 | 724 | 41 | -0.89 | 0.000131 |
| AK3 | 25.6 | 227 | 43 | 1.02 | 0.000135 |
| EEF2 | 95.3 | 858 | 32 | -0.94 | 0.000135 |
| S100A11 | 11.7 | 105 | 39 | -0.86 | 0.000156 |
| CALU | 37.1 | 315 | 63 | 0.72 | 0.000156 |
| TUBB4B | 49.8 | 445 | 44 | -1.23 | 0.000177 |
| CAV1 | 20.5 | 178 | 51 | 0.72 | 0.00018 |
| MCFD2 | 16.4 | 146 | 45 | 1.13 | 0.000181 |
| RPS16 | 16.4 | 146 | 47 | -0.42 | 0.000185 |
| YWHAQ | 27.7 | 245 | 40 | -1.12 | 0.000193 |
| MARCKS | 31.5 | 332 | 61 | -0.79 | 0.000197 |
| EIF5A | 16.8 | 154 | 41 | -1.1 | 0.000198 |
| CLIC1 | 26.9 | 241 | 32 | -1.12 | 0.000213 |
| MYO1C | 121.6 | 1063 | 33 | 0.34 | 0.000229 |
| GLUD1 | 61.4 | 558 | 31 | 0.41 | 0.000235 |
| HSP90AA1 | 84.6 | 732 | 41 | -0.73 | 0.00024 |
| H2BC12 | 13.9 | 126 | 47 | -0.87 | 0.000242 |
| PCYOX1 | 56.6 | 505 | 34 | 0.98 | 0.000282 |
| TXNDC5 | 47.6 | 432 | 37 | 0.73 | 0.000284 |
| FLOT2 | 47 | 428 | 32 | 0.52 | 0.000304 |
| HYOU1 | 111.3 | 999 | 32 | 0.57 | 0.000305 |
| RPN2 | 69.2 | 631 | 41 | 0.66 | 0.00033 |
| PSME1 | 28.7 | 249 | 36 | -0.87 | 0.000383 |
| ACADVL | 70.3 | 655 | 34 | 0.78 | 0.000387 |
| RAB1A | 22.7 | 205 | 60 | 0.57 | 0.000463 |
| TMED9 | 27.3 | 235 | 34 | 0.43 | 0.00048 |
| CAVIN3 | 27.7 | 261 | 43 | 0.27 | 0.000499 |
| PGRMC1 | 21.7 | 195 | 31 | 0.36 | 0.000539 |
| TXNDC12 | 19.2 | 172 | 33 | 0.81 | 0.000595 |
| H2AX | 15.1 | 143 | 33 | -0.76 | 0.00062 |
| DYNLRB1 | 10.9 | 96 | 34 | -0.43 | 0.000627 |
| NENF | 18.8 | 172 | 38 | 0.79 | 0.000692 |
| TPI1 | 26.7 | 249 | 51 | -1.12 | 0.000713 |
| NIBAN2 | 84.1 | 746 | 39 | -0.7 | 0.000786 |
| HSPA1B | 70 | 641 | 44 | -0.71 | 0.000789 |
| ARL8B | 21.5 | 186 | 35 | -0.59 | 0.000789 |
| UBE2N | 17.1 | 152 | 37 | -1.13 | 0.000798 |
| RRAS | 23.5 | 218 | 33 | 0.63 | 0.00084 |
| RPS3 | 26.7 | 243 | 51 | -0.55 | 0.000858 |
| SH3BGRL3 | 10.4 | 93 | 31 | -0.94 | 0.000906 |
| SSBP1 | 17.2 | 148 | 38 | 0.6 | 0.000977 |
| RAB5C | 23.5 | 216 | 48 | 0.48 | 0.000989 |
| MTPN | 12.9 | 118 | 35 | -0.99 | 0.001085 |
| CALR | 48.1 | 417 | 69 | 0.72 | 0.001121 |
| HSPA5 | 72.3 | 654 | 50 | 0.41 | 0.001133 |
| RAB14 | 23.9 | 215 | 48 | 0.31 | 0.001195 |
| MYL9 | 19.8 | 172 | 51 | -0.78 | 0.001337 |
| ANXA5 | 35.9 | 320 | 66 | -0.97 | 0.001469 |
| EHD1 | 60.6 | 534 | 41 | -0.85 | 0.001565 |
| MYL6 | 16.9 | 151 | 52 | -0.59 | 0.001653 |
| MYL12B | 19.8 | 172 | 51 | -0.67 | 0.001746 |
| PGK1 | 44.6 | 417 | 39 | -0.82 | 0.001928 |
| RPS15A | 14.8 | 130 | 36 | -0.27 | 0.00195 |
| VAMP2 | 12.7 | 116 | 34 | 0.27 | 0.002233 |
| PRKCSH | 59.4 | 528 | 33 | 0.38 | 0.002542 |
| IFITM3 | 14.6 | 133 | 31 | -0.48 | 0.002635 |
| RCN2 | 36.9 | 317 | 43 | 0.61 | 0.002888 |
| ARF3 | 20.6 | 181 | 54 | -0.53 | 0.003277 |
| CNN1 | 33.2 | 297 | 39 | 0.27 | 0.003631 |
| PARK7 | 19.9 | 189 | 44 | -0.91 | 0.004275 |
| COX5A | 16.8 | 150 | 47 | 0.59 | 0.004365 |
| DBI | 10 | 87 | 51 | -1.43 | 0.004454 |
| ANXA4 | 35.9 | 319 | 41 | -0.51 | 0.004561 |
| SND1 | 101.9 | 910 | 33 | -0.54 | 0.004709 |
| RAB11B | 24.5 | 218 | 32 | 0.36 | 0.005453 |
| P4HA2 | 60.9 | 535 | 40 | 0.51 | 0.005815 |
| PDIA3 | 56.7 | 505 | 55 | 0.43 | 0.006018 |
| LRPAP1 | 41.4 | 357 | 35 | 0.53 | 0.006428 |
| CYB5A | 15.3 | 134 | 36 | 0.72 | 0.006808 |
| PLOD1 | 83.5 | 727 | 31 | 0.58 | 0.007065 |
| RPS3A | 29.9 | 264 | 33 | -0.58 | 0.009505 |
| GANAB | 106.8 | 944 | 36 | 0.61 | 0.009988 |
| RPS8 | 24.2 | 208 | 33 | -0.38 | 0.010023 |
| BAG2 | 23.8 | 211 | 39 | 0.41 | 0.01005 |
| CCT2 | 57.5 | 535 | 34 | -0.43 | 0.013506 |
| RAP1B | 20.8 | 184 | 34 | -0.37 | 0.01363 |
| PGAM1 | 28.8 | 254 | 55 | -0.54 | 0.016202 |
| H2AZ1 | 13.5 | 128 | 54 | -0.66 | 0.017084 |
| MAP4 | 120.9 | 1152 | 30 | -0.61 | 0.017349 |
| YWHAG | 28.3 | 247 | 36 | -1.05 | 0.018193 |
| PLEC | 531.5 | 4684 | 48 | 0.19 | 0.018786 |
| ELOC | 12.5 | 112 | 37 | -0.45 | 0.023803 |
| PDLIM7 | 49.8 | 457 | 42 | -0.61 | 0.025568 |
| VAMP3 | 11.3 | 100 | 40 | 0.61 | 0.029384 |
| DDOST | 50.8 | 456 | 41 | 0.54 | 0.029628 |
| ANPEP | 109.5 | 967 | 41 | 0.42 | 0.032193 |
| CYCS | 11.7 | 105 | 41 | 0.66 | 0.0374 |
| ARL1 | 20.4 | 181 | 44 | 0.76 | 0.038062 |
| EEF1A1 | 50.1 | 462 | 32 | -0.68 | 0.039306 |
| LMNB1 | 66.4 | 586 | 34 | -0.79 | 0.039443 |
| STOM | 31.7 | 288 | 46 | 0.33 | 0.04317 |
| RPS19 | 16.1 | 145 | 53 | -0.41 | 0.044036 |
| Etoposide |  |  |  |  |  |
| Name | KDa | N° AAs | Coverage | Log 2 Ratio | P-value |
| UBE2L3 | 17.9 | 154 | 46 | -1.91 | 2.98E-08 |
| MYH10 | 228.9 | 1976 | 36 | -1.22 | 3.73E-08 |
| ATP5PD | 18.5 | 161 | 41 | 1.07 | 4.16E-08 |
| COX4I1 | 19.6 | 169 | 38 | 0.71 | 1.32E-07 |
| NT5E | 63.3 | 574 | 38 | 1.16 | 1.06E-06 |
| DPYSL2 | 62.3 | 572 | 55 | -1.14 | 2.19E-06 |
| H2AZ1 | 13.5 | 128 | 54 | -1.26 | 2.47E-06 |
| H2AX | 15.1 | 143 | 33 | -1.15 | 2.61E-06 |
| H4C1 | 11.4 | 103 | 51 | -1.18 | 2.95E-06 |
| H2BC12 | 13.9 | 126 | 47 | -1.13 | 3.83E-06 |
| ALDH1A1 | 54.8 | 501 | 40 | -1.62 | 4.04E-06 |
| VDAC1 | 30.8 | 283 | 57 | 0.93 | 4.36E-06 |
| VIM | 53.6 | 466 | 80 | 0.78 | 4.49E-06 |
| PDLIM7 | 49.8 | 457 | 42 | -1 | 4.79E-06 |
| LMNB1 | 66.4 | 586 | 34 | -1.2 | 5.47E-06 |
| DPYSL3 | 61.9 | 570 | 49 | -1.66 | 6.85E-06 |
| HADHB | 51.3 | 474 | 32 | 0.89 | 8.89E-06 |
| SQOR | 49.9 | 450 | 45 | 1.02 | 1.26E-05 |
| ETFA | 35.1 | 333 | 39 | 1.18 | 1.28E-05 |
| SEPTIN7 | 50.6 | 437 | 35 | -0.48 | 1.49E-05 |
| UBE2N | 17.1 | 152 | 37 | -1.19 | 1.67E-05 |
| ATP1A1 | 112.8 | 1023 | 31 | 0.56 | 1.81E-05 |
| ACADVL | 70.3 | 655 | 34 | 1.11 | 3.53E-05 |
| HADHA | 82.9 | 763 | 43 | 0.84 | 8.43E-05 |
| MARCKS | 31.5 | 332 | 61 | -0.8 | 8.71E-05 |
| FERMT2 | 77.8 | 680 | 33 | -0.71 | 8.78E-05 |
| ATP5F1D | 17.5 | 168 | 38 | 0.83 | 9.74E-05 |
| ATP5ME | 7.9 | 69 | 32 | 0.73 | 9.96E-05 |
| SEPTIN2 | 41.5 | 361 | 49 | -0.55 | 0.000111 |
| ATP5F1A | 59.7 | 553 | 44 | 0.7 | 0.000124 |
| HMGA2 | 11.8 | 109 | 31 | 1.06 | 0.000147 |
| XRCC6 | 69.8 | 609 | 35 | -0.57 | 0.000197 |
| NES | 177.3 | 1621 | 34 | 0.83 | 0.000218 |
| TUFM | 49.8 | 455 | 32 | 0.94 | 0.000232 |
| UGDH | 55 | 494 | 31 | -0.81 | 0.000243 |
| TAGLN | 22.6 | 201 | 58 | -1.02 | 0.000247 |
| VDAC3 | 30.6 | 283 | 32 | 0.44 | 0.000256 |
| CKAP4 | 66 | 602 | 56 | 0.91 | 0.00027 |
| H2BC11 | 13.9 | 126 | 47 | -1.66 | 0.000322 |
| ERLIN2 | 37.8 | 339 | 41 | 0.83 | 0.000324 |
| TAGLN2 | 22.4 | 199 | 56 | -1.04 | 0.000347 |
| VAMP2 | 12.7 | 116 | 34 | 0.54 | 0.000411 |
| GSTK1 | 25.5 | 226 | 33 | 0.97 | 0.000449 |
| TLN1 | 269.6 | 2541 | 45 | -0.74 | 0.000464 |
| MLEC | 32.2 | 292 | 37 | 0.74 | 0.000509 |
| FSCN1 | 54.5 | 493 | 30 | -1.04 | 0.000546 |
| IER3IP1 | 9 | 82 | 34 | 0.48 | 0.000602 |
| CNPY2 | 20.6 | 182 | 37 | 1.19 | 0.000621 |
| PDLIM1 | 36 | 329 | 38 | -0.73 | 0.000637 |
| APMAP | 46.5 | 416 | 37 | 0.86 | 0.000722 |
| IFITM3 | 14.6 | 133 | 31 | -0.65 | 0.00078 |
| HSPA9 | 73.6 | 679 | 47 | 0.71 | 0.000828 |
| AKAP12 | 191.4 | 1782 | 31 | -0.61 | 0.000912 |
| MYL9 | 19.8 | 172 | 51 | -0.88 | 0.00099 |
| ATP5PF | 12.6 | 108 | 31 | 0.61 | 0.001005 |
| RPL7 | 29.2 | 248 | 40 | 0.35 | 0.001235 |
| H2AC4 | 14.1 | 130 | 33 | -0.9 | 0.001317 |
| ACTN4 | 104.8 | 911 | 56 | -0.54 | 0.001394 |
| ETFB | 27.8 | 255 | 36 | 0.92 | 0.001396 |
| AK3 | 25.6 | 227 | 43 | 1.38 | 0.001423 |
| KRT18 | 48 | 430 | 49 | 1.43 | 0.001796 |
| LAMTOR1 | 17.7 | 161 | 48 | 0.46 | 0.001821 |
| CNN1 | 33.2 | 297 | 39 | -0.51 | 0.001823 |
| VCL | 123.7 | 1134 | 54 | -0.8 | 0.002265 |
| SEPTIN11 | 49.4 | 429 | 33 | -0.28 | 0.002645 |
| SOD2 | 24.7 | 222 | 63 | 1.23 | 0.002779 |
| HSD17B12 | 34.3 | 312 | 36 | 0.89 | 0.002998 |
| CYB5R3 | 34.2 | 301 | 53 | 0.52 | 0.003306 |
| RPS15 | 17 | 145 | 55 | -0.52 | 0.003391 |
| MYL12B | 19.8 | 172 | 51 | -0.61 | 0.004723 |
| RRBP1 | 152.4 | 1410 | 50 | 0.6 | 0.004869 |
| IKBIP | 39.3 | 350 | 37 | 0.97 | 0.006382 |
| ASPH | 85.8 | 758 | 32 | 0.54 | 0.007688 |
| PHB2 | 33.3 | 299 | 38 | 0.44 | 0.007977 |
| NAPA | 33.2 | 295 | 34 | 0.54 | 0.008259 |
| TPM4 | 28.5 | 248 | 44 | -0.65 | 0.008463 |
| CANX | 67.5 | 592 | 45 | 0.6 | 0.008745 |
| XRCC5 | 82.7 | 732 | 40 | -0.56 | 0.009069 |
| MYL6 | 16.9 | 151 | 52 | -0.51 | 0.009312 |
| ARL1 | 20.4 | 181 | 44 | 0.62 | 0.010019 |
| GDI2 | 50.6 | 445 | 49 | -0.68 | 0.010448 |
| FLNA | 280.6 | 2647 | 52 | -0.6 | 0.010505 |
| ATP5F1B | 56.5 | 529 | 67 | 0.65 | 0.010573 |
| ANXA6 | 75.8 | 673 | 55 | -0.74 | 0.010774 |
| TCP1 | 60.3 | 556 | 31 | -0.42 | 0.011113 |
| ACAT1 | 45.2 | 427 | 35 | 0.46 | 0.011448 |
| TUBB4B | 49.8 | 445 | 44 | -0.72 | 0.011516 |
| TUBB | 49.6 | 444 | 44 | -0.69 | 0.012889 |
| LASP1 | 29.7 | 261 | 37 | -0.71 | 0.013486 |
| EIF5A | 16.8 | 154 | 41 | -0.69 | 0.014173 |
| MYOF | 234.6 | 2061 | 43 | 0.33 | 0.01439 |
| MYH9 | 226.4 | 1960 | 50 | -0.5 | 0.014889 |
| TPM1 | 32.7 | 284 | 38 | -0.49 | 0.015645 |
| TPM2 | 32.8 | 284 | 38 | -0.51 | 0.015658 |
| RAN | 24.4 | 216 | 41 | -0.59 | 0.016301 |
| ENO1 | 47.1 | 434 | 53 | -0.81 | 0.017036 |
| CAVIN1 | 43.5 | 390 | 37 | 0.36 | 0.018315 |
| SSR4 | 19 | 173 | 31 | 0.6 | 0.018396 |
| HSPB1 | 22.8 | 205 | 77 | -0.62 | 0.019225 |
| DBI | 10 | 87 | 51 | -1.04 | 0.020016 |
| MME | 85.5 | 750 | 35 | -0.53 | 0.020641 |
| PGRMC2 | 23.8 | 223 | 32 | 0.62 | 0.02231 |
| ANPEP | 109.5 | 967 | 41 | 0.44 | 0.022319 |
| IMMT | 83.6 | 758 | 32 | 0.47 | 0.025374 |
| AKR1B1 | 35.8 | 316 | 45 | 0.66 | 0.028248 |
| TKT | 67.8 | 623 | 40 | -0.52 | 0.029598 |
| P4HB | 57.1 | 508 | 67 | 0.91 | 0.030145 |
| RAB14 | 23.9 | 215 | 48 | 0.31 | 0.031213 |
| OAT | 48.5 | 439 | 38 | 0.81 | 0.034236 |
| RAP1B | 20.8 | 184 | 34 | -0.32 | 0.034289 |
| S100A13 | 11.5 | 98 | 42 | 0.4 | 0.035209 |
| PEA15 | 15 | 130 | 49 | -1.34 | 0.035378 |
| HSD17B10 | 26.9 | 261 | 49 | 0.45 | 0.036197 |
| PHB1 | 29.8 | 272 | 49 | 0.37 | 0.037735 |
| SEC22B | 24.7 | 215 | 50 | 0.55 | 0.037739 |
| TUBB6 | 49.8 | 446 | 49 | -0.5 | 0.044301 |

**Supplementary Table 13|** Reactome pathway analysis that determines whether certain pathways are enriched in the submitted protein data set. The analysis is based on a statistical hypergeometric distribution test. The test produces a probability score, which is corrected for a false discovery rate (FDR) using the Benjamini-Hochberg method.

| Cell passage |  |  |  |
| --- | --- | --- | --- |
| Pathway name | Entities | Reactions | FDR |
| Gene and protein expression by JAK-STAT signaling after Interleukin 12 stimulation | 0.025 | 0.003 | 0.021 |
| Interleukin 12 signaling | 0.025 | 0.004 | 0.021 |
| Interleukin 12 family signaling | 0.026 | 0.008 | 0.021 |
| Cellular response to heat stress | 0.021 | 0.002 | 0.021 |
| HSF1 dependent transactivation | 0.021 | 5.64E-04 | 0.021 |
| Attenuation phase | 0.021 | 3.52E-04 | 0.021 |
| Regulation of HSF1 mediated heat shock response | 0.021 | 9.86E-04 | 0.021 |
| HSF1 activation | 0.021 | 4.93E-04 | 0.021 |
| Signaling by interleukins | 0.12 | 0.04 | 0.03 |
| Irradiation |  |  |  |
| Pathway name | Entities | Reactions | FDR |
| Gene and protein expression by JAK-STAT signaling after Interleukin 12 stimulation | 0.025 | 0.003 | 0.008 |
| Interleukin 12 signaling | 0.025 | 0.004 | 0.008 |
| Interleukin 12 family signaling | 0.026 | 0.008 | 0.008 |
| Signaling by Interleukins | 0.12 | 0.036 | 0.132 |
| Cytokine signaling in immune system | 0.185 | 0.052 | 0.421 |
| Immune system | 0.198 | 0.117 | 0.429 |
| Organelle biogenesis and maintenance | 0.012 | 0.006 | 0.485 |
| Mitochondria biogenesis | 0.012 | 0.003 | 0.485 |
| Transcriptional activation of mitochondrial biogenesis | 0.012 | 0.002 | 0.485 |
| Hydroxyurea |  |  |  |
| Pathway name | Entities | Reactions | FDR |
| Interleukin 12 signaling | 0.025 | 0.004 | 0.014 |
| Gene and protein expression by JAK-STAT signaling after Interleukin 12 stimulation | 0.025 | 0.003 | 0.014 |
| Interleukin 12 family signaling | 0.026 | 0.008 | 0.014 |
| Cellular response to heat stress | 0.021 | 0.002 | 0.014 |
| HSF1 dependent transactivation | 0.021 | 5.64E-04 | 0.014 |
| Attenuation phase | 0.021 | 3.52E-04 | 0.014 |
| Regulation of HSF1 mediated heat shock response | 0.021 | 9.86E-04 | 0.014 |
| HSF1 activation | 0.021 | 4.93E-04 | 0.014 |
| Cellular response to stress | 0.1 | 0.032 | 0.039 |
| Etoposide |  |  |  |
| Pathway name | Entities | Reactions | FDR |
| Interleukin 12 signaling | 0.025 | 0.004 | 0.009 |
| Gene and protein expression by JAK-STAT signaling after Interleukin 12 stimulation | 0.025 | 0.003 | 0.009 |
| Interleukin 12 family signaling | 0.026 | 0.008 | 0.009 |
| Signaling by interleukins | 0.12 | 0.0036 | 0.016 |
| Regulation of Nf-kappa B signaling | 6.84E-04 | 7.75E-04 | 0.016 |
| TAK1-dependent IKK and Nf-kappa-B activation | 6.84E-04 | 0.001 | 0.016 |
| MyD88 cascade initiated on plasma membrane | 6.84E-04 | 0.005 | 0.016 |
| Toll Like Receptor 10 (TLR10) cascade | 6.84E-04 | 0.005 | 0.016 |
| Toll Like Receptor 5 (TLR5) cascade | 6.84E-04 | 0.005 | 0.016 |

**Supplementary Table 14|** Pathways analysis of cells at passage 20, cells treated with 12 Gy irradiation, 800 µM hydroxyurea and 10 µM etoposide. This analysis has been performed through MetaboAnalysist by joining significant metabolomics and proteomics data outputs. The analysis is based on a statistical hypergeometric distribution test and betweenness centrality measure.

| Cell Passage |  |  |  |
| --- | --- | --- | --- |
| **Pathway Name** | **p value** | **FDR** | **Impact** |
| [Glycolysis or Gluconeogenesis](https://www.metaboanalyst.ca/MetaboAnalyst/Secure/pathinteg/IntegResultView.xhtml) | 6.97E-12 | 5.86E-10 | 1.4877 |
| [Pyruvate metabolism](https://www.metaboanalyst.ca/MetaboAnalyst/Secure/pathinteg/IntegResultView.xhtml) | 3.18E-04 | 0.013343 | 0.16173 |
| [Fructose and mannose metabolism](https://www.metaboanalyst.ca/MetaboAnalyst/Secure/pathinteg/IntegResultView.xhtml) | 0.017186 | 0.29291 | 0.31658 |
| [Aminoacyl-tRNA biosynthesis](https://www.metaboanalyst.ca/MetaboAnalyst/Secure/pathinteg/IntegResultView.xhtml) | 0.018158 | 0.29291 | 0 |
| [Taurine and hypotaurine metabolism](https://www.metaboanalyst.ca/MetaboAnalyst/Secure/pathinteg/IntegResultView.xhtml) | 0.020018 | 0.29291 | 0.057143 |
| [Amino sugar and nucleotide sugar metabolism](https://www.metaboanalyst.ca/MetaboAnalyst/Secure/pathinteg/IntegResultView.xhtml) | 0.022575 | 0.29291 | 0.07709 |
| [Pentose phosphate pathway](https://www.metaboanalyst.ca/MetaboAnalyst/Secure/pathinteg/IntegResultView.xhtml) | 0.026401 | 0.29291 | 0.91291 |
| [Propanoate metabolism](https://www.metaboanalyst.ca/MetaboAnalyst/Secure/pathinteg/IntegResultView.xhtml) | 0.027896 | 0.29291 | 0.072618 |
| [Glutathione metabolism](https://www.metaboanalyst.ca/MetaboAnalyst/Secure/pathinteg/IntegResultView.xhtml) | 0.041465 | 0.38701 | 0.29331 |
| Irradiation |  |  |  |
| **Pathway Name** | **p value** | **FDR** | **Impact** |
| [Glycolysis or Gluconeogenesis](https://www.metaboanalyst.ca/MetaboAnalyst/Secure/pathinteg/IntegResultView.xhtml) | 4.97E-05 | 0.004174 | 0.62439 |
| [Aminoacyl-tRNA biosynthesis](https://www.metaboanalyst.ca/MetaboAnalyst/Secure/pathinteg/IntegResultView.xhtml) | 1.74E-04 | 0.007326 | 0 |
| [Pyruvate metabolism](https://www.metaboanalyst.ca/MetaboAnalyst/Secure/pathinteg/IntegResultView.xhtml) | 0.006217 | 0.13662 | 0.16173 |
| [Arginine and proline metabolism](https://www.metaboanalyst.ca/MetaboAnalyst/Secure/pathinteg/IntegResultView.xhtml) | 0.008928 | 0.13662 | 0.12286 |
| [Arginine biosynthesis](https://www.metaboanalyst.ca/MetaboAnalyst/Secure/pathinteg/IntegResultView.xhtml) | 0.009738 | 0.13662 | 0.14615 |
| [Nitrogen metabolism](https://www.metaboanalyst.ca/MetaboAnalyst/Secure/pathinteg/IntegResultView.xhtml) | 0.011385 | 0.13662 | 0 |
| [D-Glutamine and D-glutamate metabolism](https://www.metaboanalyst.ca/MetaboAnalyst/Secure/pathinteg/IntegResultView.xhtml) | 0.011385 | 0.13662 | 0.055556 |
| [Phenylalanine, tyrosine and tryptophan biosynthesis](https://www.metaboanalyst.ca/MetaboAnalyst/Secure/pathinteg/IntegResultView.xhtml) | 0.013768 | 0.14457 | 0.62222 |
| [Histidine metabolism](https://www.metaboanalyst.ca/MetaboAnalyst/Secure/pathinteg/IntegResultView.xhtml) | 0.015582 | 0.14543 | 0.097849 |
| [Taurine and hypotaurine metabolism](https://www.metaboanalyst.ca/MetaboAnalyst/Secure/pathinteg/IntegResultView.xhtml) | 0.02849 | 0.23931 | 0.057143 |
| [Nicotinate and nicotinamide metabolism](https://www.metaboanalyst.ca/MetaboAnalyst/Secure/pathinteg/IntegResultView.xhtml) | 0.032128 | 0.24534 | 0.2037 |
| [beta-Alanine metabolism](https://www.metaboanalyst.ca/MetaboAnalyst/Secure/pathinteg/IntegResultView.xhtml) | 0.03622 | 0.25354 | 0.013289 |
| [Propanoate metabolism](https://www.metaboanalyst.ca/MetaboAnalyst/Secure/pathinteg/IntegResultView.xhtml) | 0.045175 | 0.28379 | 0.072618 |
| Hydroxyurea |  |  |  |
| **Pathway Name** | **p value** | **FDR** | **Impact** |
| [Glutathione metabolism](https://www.metaboanalyst.ca/MetaboAnalyst/Secure/pathinteg/IntegResultView.xhtml) | 8.13E-05 | 0.005958 | 0.60414 |
| [Glycolysis or Gluconeogenesis](https://www.metaboanalyst.ca/MetaboAnalyst/Secure/pathinteg/IntegResultView.xhtml) | 1.42E-04 | 0.005958 | 0.48117 |
| [Nitrogen metabolism](https://www.metaboanalyst.ca/MetaboAnalyst/Secure/pathinteg/IntegResultView.xhtml) | 7.79E-04 | 0.016351 | 0.083333 |
| [D-Glutamine and D-glutamate metabolism](https://www.metaboanalyst.ca/MetaboAnalyst/Secure/pathinteg/IntegResultView.xhtml) | 7.79E-04 | 0.016351 | 0.11111 |
| [Pyruvate metabolism](https://www.metaboanalyst.ca/MetaboAnalyst/Secure/pathinteg/IntegResultView.xhtml) | 0.001613 | 0.023243 | 0.083333 |
| [Arginine biosynthesis](https://www.metaboanalyst.ca/MetaboAnalyst/Secure/pathinteg/IntegResultView.xhtml) | 0.00166 | 0.023243 | 0.38769 |
| [Aminoacyl-tRNA biosynthesis](https://www.metaboanalyst.ca/MetaboAnalyst/Secure/pathinteg/IntegResultView.xhtml) | 0.002832 | 0.033978 | 0 |
| [Arginine and proline metabolism](https://www.metaboanalyst.ca/MetaboAnalyst/Secure/pathinteg/IntegResultView.xhtml) | 0.003699 | 0.038837 | 0.1461 |
| [Propanoate metabolism](https://www.metaboanalyst.ca/MetaboAnalyst/Secure/pathinteg/IntegResultView.xhtml) | 0.013603 | 0.12265 | 0.078168 |
| [Lysine degradation](https://www.metaboanalyst.ca/MetaboAnalyst/Secure/pathinteg/IntegResultView.xhtml) | 0.014602 | 0.12265 | 0.050975 |
| [Butanoate metabolism](https://www.metaboanalyst.ca/MetaboAnalyst/Secure/pathinteg/IntegResultView.xhtml) | 0.01824 | 0.13929 | 0.13889 |
| [Glyoxylate and dicarboxylate metabolism](https://www.metaboanalyst.ca/MetaboAnalyst/Secure/pathinteg/IntegResultView.xhtml) | 0.022898 | 0.16029 | 0.005724 |
| [Valine, leucine and isoleucine degradation](https://www.metaboanalyst.ca/MetaboAnalyst/Secure/pathinteg/IntegResultView.xhtml) | 0.027483 | 0.17758 | 0.23621 |
| Etoposide |  |  |  |
| **Pathway Name** | **p value** | **FDR** | **Impact** |
| [Valine, leucine and isoleucine degradation](https://www.metaboanalyst.ca/MetaboAnalyst/Secure/pathinteg/IntegResultView.xhtml) | 1.89E-04 | 0.015884 | 0.26415 |
| [Valine, leucine and isoleucine biosynthesis](https://www.metaboanalyst.ca/MetaboAnalyst/Secure/pathinteg/IntegResultView.xhtml) | 0.006248 | 0.26242 | 0 |
| [Lysine degradation](https://www.metaboanalyst.ca/MetaboAnalyst/Secure/pathinteg/IntegResultView.xhtml) | 0.012834 | 0.35934 | 0.049645 |
| [Fatty acid degradation](https://www.metaboanalyst.ca/MetaboAnalyst/Secure/pathinteg/IntegResultView.xhtml) | 0.018502 | 0.38854 | 1.613 |
| [Butanoate metabolism](https://www.metaboanalyst.ca/MetaboAnalyst/Secure/pathinteg/IntegResultView.xhtml) | 0.03456 | 0.47271 | 0.13889 |
| [Fatty acid elongation](https://www.metaboanalyst.ca/MetaboAnalyst/Secure/pathinteg/IntegResultView.xhtml) | 0.039487 | 0.47271 | 1.599 |
| [Pentose and glucuronate interconversions](https://www.metaboanalyst.ca/MetaboAnalyst/Secure/pathinteg/IntegResultView.xhtml) | 0.041441 | 0.47271 | 0.073118 |
| [Amino sugar and nucleotide sugar metabolism](https://www.metaboanalyst.ca/MetaboAnalyst/Secure/pathinteg/IntegResultView.xhtml) | 0.04502 | 0.47271 | 0.014652 |


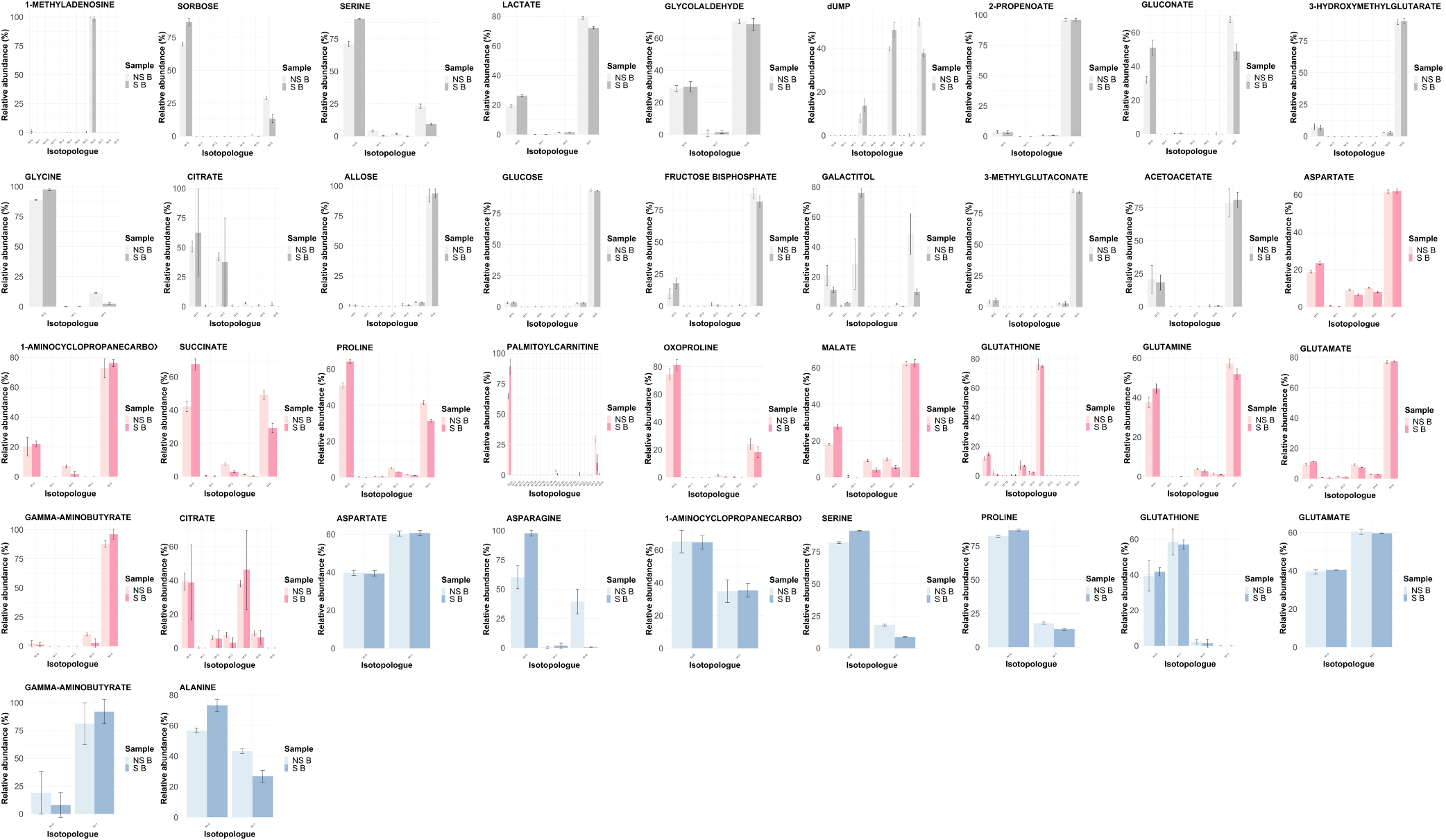


**Supplementary Figure 8|** Relative abundance of labeled isotopes incorporation in the metabolites identified for passaged cells. the [^13^C_6_]-glucose in black, and [^13^C_5_]-glutamine in red, and [^15^N_2_]-glutamine in blue. NS=non-senescence, S=senescence.


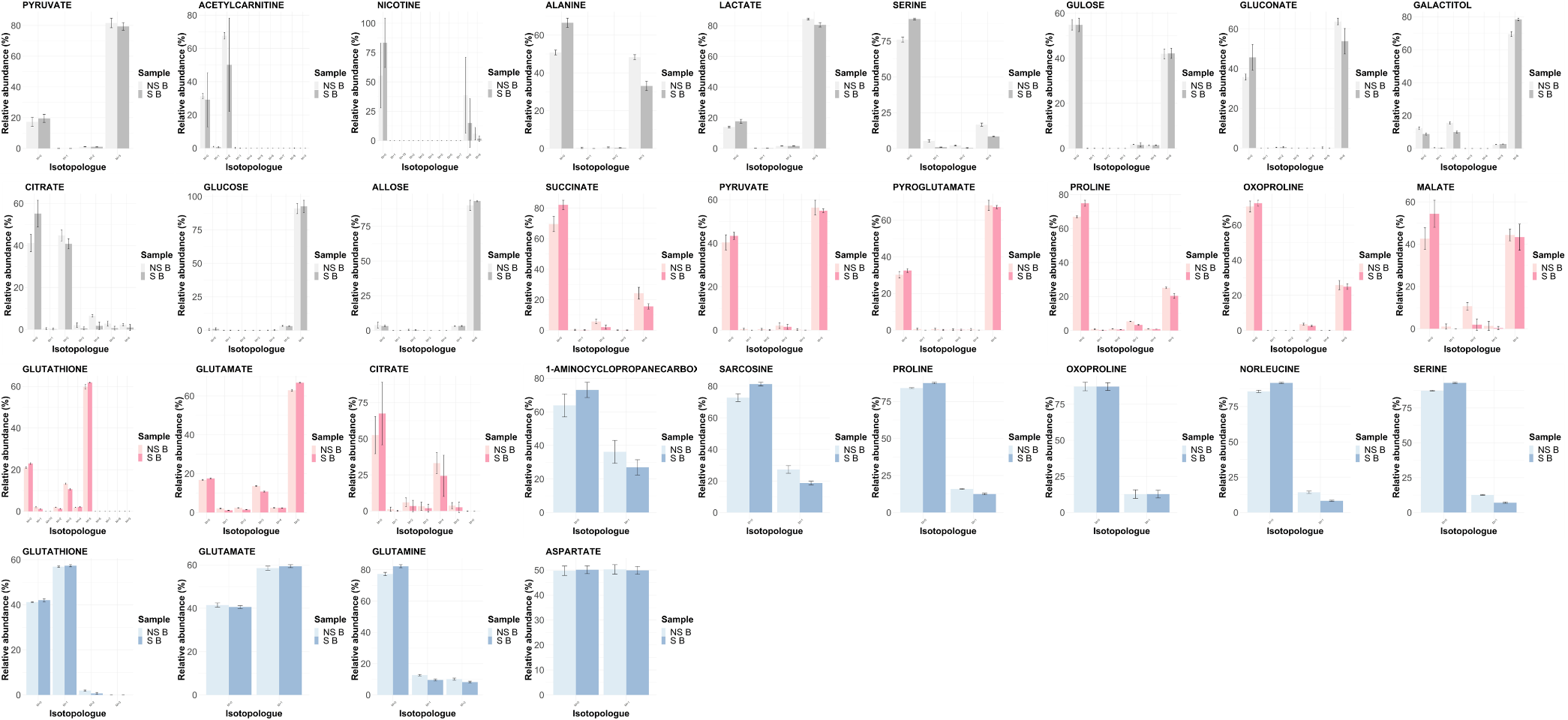


**Supplementary Figure 9|** Relative abundance of labeled isotopes incorporation in the metabolites identified for irradiated cells. the [^13^C_6_]-glucose in black, and [^13^C_5_]-glutamine in red, and [^15^N_2_]-glutamine in blue. NS=non-senescence, S=senescence.


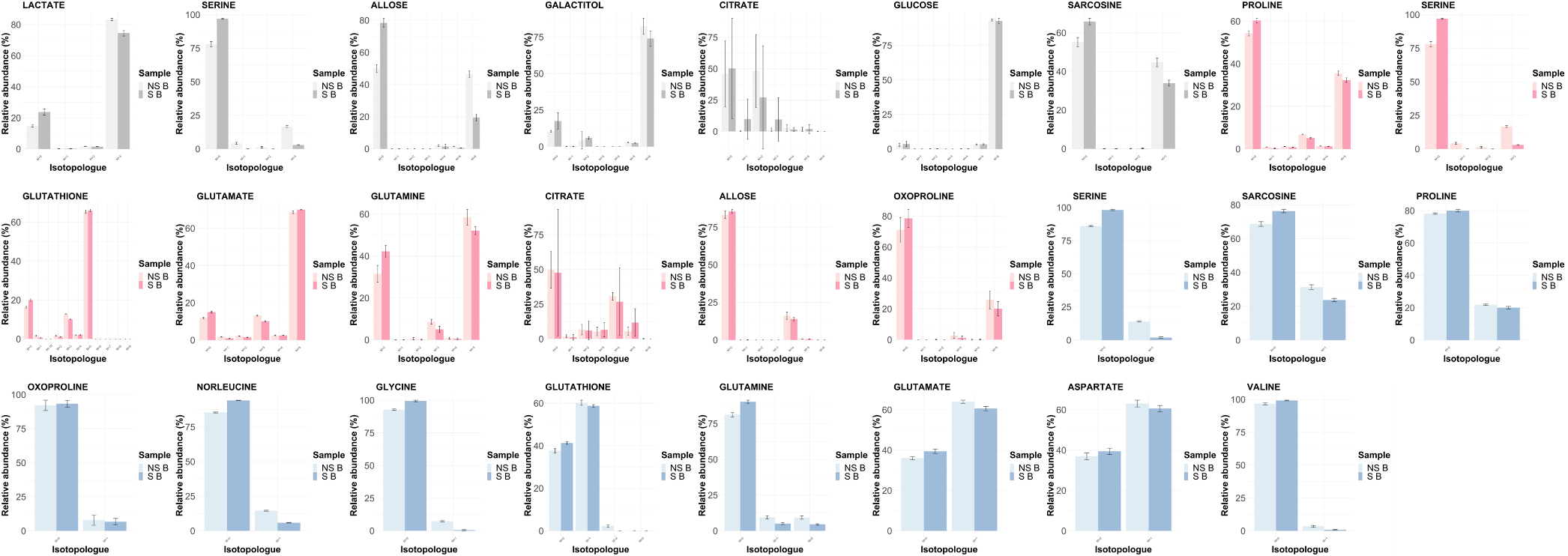


**Supplementary Figure 10|** Relative abundance of labeled isotopes incorporation in the metabolites identified for hydroxyurea-treated cells. the [^13^C_6_]-glucose in black, and [^13^C_5_]-glutamine in red, and [^15^N_2_]-glutamine in blue. NS=non-senescence, S=senescence.


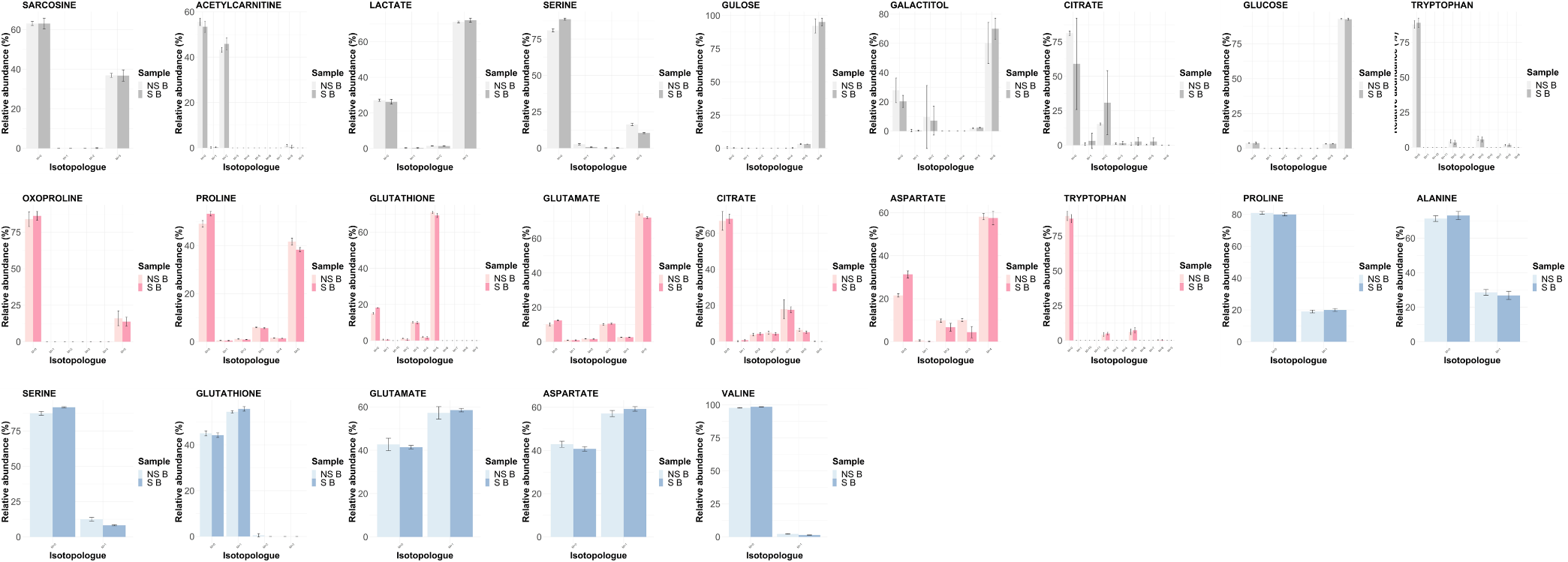


**Supplementary Figure 1|** Relative abundance of labeled isotopes incorporation in the metabolites identified for etoposide-treated cells. the [^13^C_6_]-glucose in black, and [^13^C_5_]-glutamine in red, and [^15^N_2_]-glutamine in blue. NS=non-senescence, S=senescence.
